# Genome-wide identification and characterisation of *R2R3-MYB* genes in sugar beet (*Beta vulgaris*)

**DOI:** 10.1186/s12870-014-0249-8

**Published:** 2014-09-25

**Authors:** Ralf Stracke, Daniela Holtgräwe, Jessica Schneider, Boas Pucker, Thomas Rosleff Sörensen, Bernd Weisshaar

**Affiliations:** Chair of Genome Research, Faculty of Biology and Center for Biotechnology, Bielefeld University, Bielefeld, 33615 Germany

**Keywords:** *Beta vulgaris*, Caryophyllales, R2R3-MYB, Transcription factor, Gene family, Flavonol regulator

## Abstract

**Background:**

The *R2R3-MYB* genes comprise one of the largest transcription factor gene families in plants, playing regulatory roles in plant-specific developmental processes, metabolite accumulation and defense responses. Although genome-wide analysis of this gene family has been carried out in some species, the *R2R3-MYB* genes in *Beta vulgaris* ssp. *vulgaris* (sugar beet) as the first sequenced member of the order Caryophyllales, have not been analysed heretofore.

**Results:**

We present a comprehensive, genome-wide analysis of the *MYB* genes from *Beta vulgaris* ssp. *vulgaris* (sugar beet) which is the first species of the order Caryophyllales with a sequenced genome. A total of 70 *R2R3-MYB* genes as well as genes encoding three other classes of MYB proteins containing multiple MYB repeats were identified and characterised with respect to structure and chromosomal organisation. Also, organ specific expression patterns were determined from RNA-seq data. The *R2R3-MYB* genes were functionally categorised which led to the identification of a sugar beet-specific clade with an atypical amino acid composition in the R3 domain, putatively encoding betalain regulators. The functional classification was verified by experimental confirmation of the prediction that the *R2R3-MYB* gene *Bv_iogq* encodes a flavonol regulator.

**Conclusions:**

This study provides the first step towards cloning and functional dissection of the role of *MYB* transcription factor genes in the nutritionally and evolutionarily interesting species *B. vulgaris*. In addition, it describes the flavonol regulator BvMYB12, being the first sugar beet R2R3-MYB with an experimentally proven function.

**Electronic supplementary material:**

The online version of this article (doi:10.1186/s12870-014-0249-8) contains supplementary material, which is available to authorized users.

## Background

Transcriptional control of gene expression influences almost all biological processes in eukaryotic cells or organisms. Transcription factors perform this function, alone or complexed with other proteins, by activating or repressing (or both) the recruitment of RNA polymerase to specific genes. The large number and diversity of transcription factors is related to their substantial regulatory complexity [1].

MYB proteins are widely distributed in all eukaryotic organisms and constitute one of the largest transcription factor families in the plant kingdom. MYB proteins are defined by a highly conserved MYB DNA-binding domain, mostly located at the N-terminus, generally consisting of up to four imperfect amino acid sequence repeats (R) of about 52 amino acids, each forming three alpha–helices [2]. The second and third helices of each repeat build a helix–turn–helix (HTH) structure with three regularly spaced tryptophan (or hydrophobic) residues, forming a hydrophobic core [3]. The third helix of each repeat is the DNA recognition helix that makes direct contact with DNA [4]. During DNA contact, two MYB repeats are closely packed in the major groove, so that the two recognition helices bind cooperatively to the specific DNA recognition sequence motif.

MYB proteins can be divided into different classes depending on the number of adjacent repeats (one, two, three or four). The three repeats of the prototypic MYB protein c-Myb [5] are referred to as R1, R2 and R3, and repeats from other MYB proteins are named according to their similarity. Plant R1R2R3-type MYB (MYB3R) proteins have been proposed to play divergent roles in cell cycle control [6,7], similar to the functions of their animal homologs.

Most plant *MYB* genes encode R2R3-MYB class proteins, containing two repeats [2,8], which are thought to have evolved from an R1R2R3-MYB gene ancestor, by the loss of the sequences encoding the R1 repeat and subsequent expansion of the gene family [9-11]. R2R3-MYB transcription factors have a modular structure, with the N-terminal MYB domain as DNA-binding domain and an activation or repression domain usually located at the highly variable C-terminus. Components for the establishment of protein-protein interactions with other components of the eukaryotic transcriptional machinery have been detected in the N-terminal module [12-14].

Based on the conservation of the MYB domain and of common amino acid motifs in the C-terminal domains, R2R3-MYB proteins have been divided into several subgroups which often group proteins with functional relationship. The reliability of the subgroups defined on the basis of phylogenetic analysis is also supported by additional criteria, such as the gene structure and the presence and position of introns [15]. Most of these subgroups, defined first for the proteins of *A. thaliana* [2,16,17], are also present, and are sometimes expanded, in other higher plants. Comparative phylogenetic studies have identified new R2R3-MYB subgroups in other plant species for which there are no representatives in *A. thaliana* (e.g. in rice, poplar and grapevine), suggesting that these proteins might have specialised functions which were either lost in *A. thaliana* or were acquired after divergence from the last common ancestor [18-20].

As initially described in the first plant *MYB* gene family review [21], the expansion of the plant-specific *R2R3-MYB* gene family is thought to be correlated with the increase in complexity of plants, particularly in Angiosperms. Consequently, the functions of *R2R3-MYB* genes are likely associated with regulating plant-specific processes including primary and secondary metabolism, developmental processes, cell fate and identity and responses to biotic and abiotic stresses [2,17,21].

With the growing number of fully sequenced plant genomes, the identification of *R2R3-MYB* genes has increased in recent times. Based on their well conserved MYB domains, *R2R3-MYB* gene families have been annotated genome-wide in *A. thaliana* (126 members) [17], *Zea mays* (157 members) [22], *Oryza sativa* (102 members) [23], *Vitis vinifera* (117 members) [19], *Populus trichocarpa* (192 members) [20], *Glycine max* (244 members) [15], *Cucumis sativus* (55 members) [24] and *Malus x domestica* (222 members) [25]. Given the potential roles of R2R3-MYB proteins in the regulation of gene expression, secondary metabolism, and responses to environmental stresses, and that *Beta vulgaris* ssp. *vulgaris* (order Caryophyllales) is the first non-rosid, non-asterid eudicot for which the genome has been sequenced [26], it is of interest to achieve a complete identification and classification of *MYB* genes in this species with respect to the number, chromosome locations, phylogenetic relationships, conserved motifs as well as expression patterns. Particularly, since sugar beet is an important crop of the temperate climates as a source for bioethanol as well as animal feed and provides nearly 30% of the worlds annual sugar production [26].

In the present study, we describe the *R2R3-MYB* gene family by means of *in silico* analysis of the *B. vulgaris* genome sequence, in order to predict protein domain architectures, and to assess the extent of conservation and divergence between *B. vulgaris* and *A. thaliana* gene families, thus leading to a functional classification of the sugar beet *MYB* genes on the basis of phylogenetic analyses. Furthermore, RNA-seq data was used to analyse expression in different *B. vulgaris* organs and to compare expression patterns of closely grouped co-orthologs. To validate the functional classification, a candidate gene was chosen for cDNA isolation and subsequent functional analysis by transient transactivation assays and complementation of an orthologous *A. thaliana* mutant. We identified the *R2R3-MYB* gene *Bv_iogq* activating two flavonol biosynthesis enzyme promoters and complementing the flavonol-deficient *myb11 myb12 myb111* mutant, and thus encoding a functional flavonol biosynthesis regulator. Our findings provide the first step towards further investigations on the biological and molecular functions of MYB transcription factors in the economically and evolutionarily interesting species *B. vulgaris*.

## Results and discussion

The annotated genome sequence of *B. vulgaris* has recently become available. It has been obtained from the double haploid breeding line KWS2320 [26]. The sequence has been assigned to nine chromosomes and *B. vulgaris* was predicted to contain 27,421 protein-coding genes (RefBeet) in 567 Mb from which 85% are chromosomally assigned.

### Identification and genomic distribution of *B. vulgaris R2R3-MYB* genes

MYB protein coding genes in *B. vulgaris* were identified using a consensus R2R3-MYB DNA binding domain sequence as protein query in TBLASTN searches on the RefBeet genome sequence. The putative MYB sequences were manually analysed for the presence of an intact MYB domain to ensure that the gene models contained two or more (multiple) MYB repeats, and that they mapped to unique loci in the genome. We identified six *B. vulgaris MYB* (*BvMYB*) genes which had been missed in the automatic annotation [26] and two which had been annotated with incomplete open reading frames. We created a primary data set of 70 R2R3-MYB proteins and three types of atypical multiple repeat MYB proteins distantly related to the typical R2R3-MYB proteins: three R1R2R3-MYB (MYB3R) proteins, one MYB4R protein and one CDC5-like protein from the *B. vulgaris* genome (Table 1). The number of atypical multiple repeat *MYB* genes identified in *B. vulgaris* is in the same range as those reported for other plant species, with for example up to six *MYB3R* and up to two *MYB4R* and *CDC5-like* genes. However, the number of *R2R3-MYB* genes is one of the smallest among the species that have been studied (ranging from 55 in *C. sativus* to in 244 *G. max*). As discussed below, this is probably due to the absence of recent genome duplication events in *B. vulgaris*.

**Table 1:** **List of annotated** ***MYB*** **genes with two or more repeats in the** ***B. vulgaris*** **ssp.** ***vulgaris*** **(KWS2320) genome**

**Gene ID**	**Gene code**	**Chr.**	**Position on pseudochr.**		**Clade (subgroup)**	**Landmark MYB in clade**	**Functional assignment**	**Protein length [aa]**	**Exon nr.**
iquc	Bv1g001230_iquc	1	1,379,505	1,381,156	C14 (S4)	AtMYB4, HvMYB5	Metabolism	341	3
owzx	Bv1g001750_owzx	1	1,903,239	1,898,047	C8 (S3)	AtMYB58, AtMYB63	Metabolism	316	3
dwki	Bv1g002800_dwki	1	3,065,434	3,067,536	C18 (S5)	VvMYBPA	Metabolism	335	2
qxpi	Bv1g006050_qxpi	1	6,597,147	6,601,421	C3	AtLMI2	Development	303	3
ksfi	Bv1g014750_ksfi	1	32,690,615	32,692,641	C12 (S14)	SlBLIND, AtRAX1	Development	363	3
zqor	Bv1ug018140_zqor	1un	38,330,908	38,328,715	C28 (S18)	HvGAMYB, AtDUO1	Development	303	3
jxgt	Bv1ug021520_jxgt	1un	45,201,741	45,199,866	C36 (S21)	AtLOF1, AtMYB52	Devel., metab.	350	2
uksi	Bv2g023560_uksi	2	127,507	126,341	C35 (S23)			253	1
wdyc	Bv2g024650_wdyc	2	1,373,912	1,378,227	C35 (S23)			416	2
ihfg	Bv2g027580_ihfg	2	4,418,041	4,424,427	C15	FaMYB1	Metabolism	199	3
jkkr	Bv2g027795_jkkr	2	4,723,639	4,720,911	C21		(metabolism)	224	3
mxck	Bv2g027990_mxck	2	4,981,898	4,984,692	C29			286	4
xprd	Bv2g029260_xprd	2	6,275,539	6,272,224	C27	EgMYB2, PtMYB4	Metabolism	422	2
ralf	Bv2g030925_ralf	2	8,431,718	8,435,595	C21		(metabolism)	237	3
ghua	Bv2g031800_ghua	2	9,813,407	9,800,544	C37 (MYB3R)	AtMYB3R1	Cell cycle	1050	11
huqy	Bv2g039110_huqy	2	27,806,443	27,798,295	C26	AtMYB26	Development	329	2
dcmm	Bv2g040720_dcmm	2	33,641,162	33,643,072	C1 (S9)	AmMIXTA, AtNOK	differentiation	454	3
mxwz	Bv2g041120_mxwz	2	34,717,448	34,719,429	C11	AtTDF1	Development	331	3
nqis	Bv2ug047120_nqis	2un	45,488,410	45,491,366	C4 (S1)	AtMYB30	Defense	344	3
urrg	Bv3g049510_urrg	3	1,126,350	1,124,818	C9 (S2)	NtMYB1, AtMYB13	Defense	288	3
hwcc	Bv3g050090_hwcc	3	1,847,195	1,845,526	C6 (S24)			360	3
cwtt	Bv3ug070140_cwtt	3un	35,695,445	35,697,294	C29			242	3
cjuq	Bv4g071740_cjuq	4	221,407	222,747	C19	VvMYB5a	Metabolism	306	2
yruo	Bv4g073190_yruo	4	1,667,414	1,671,378	C27	EgMYB2, PtMYB4	Metabolism	397	2
ygxg	Bv4g074860_ygxg	4	3,396,314	3,392,041	C28 (S18)	HvGAMYB, AtDUO1	Development	556	3
skuh	Bv4g078900_skuh	4	7,533,943	7,547,807	C42 (MYB4R)	AtMYB4R1		919	12
zfig	Bv4g079610_zfig	4	8,546,454	8,544,269	C25 (S13)	AtMYB61	Metabolism	449	3
oref	Bv4g079670_oref	4	8,669,284	8,678,138	C41 (CDC5)	AtCDC5	Cell cycle	991	4
josh	Bv4g083815_josh	4	16,552,338	16,566,035				209	3
rwwj	Bv4g084340_rwwj	4	18,292,096	18,285,979	C14 (S4)	AtMYB4, HvMYB5	Metabolism	322	3
xwne	Bv4g091510_xwne	4	32,888,798	32,889,966	C11	AtTDF1	Development	316	3
jofq	Bv5g098940_jofq	5	834,523	837,220	C10			294	2
sskd	Bv5g100530_sskd	5	2,767,018	2,770,045	C23	AtMYB103	Development	378	3
mhxh	Bv5g101320_mhxh	5	3,800,529	3,804,868	C37 (MYB3R)	AtMYB3R1	Cell cycle	525	7
zkef	Bv5g107260_zkef	5	14,451,259	14,465,332				272	2
ztyd	Bv5g110930_ztyd	5	25,553,310	25,538,334	C32 (S19)	AtMYB21	Development	231	3
udmh	Bv5g110960_udmh	5	25,780,619	25,782,807	C4 (S1)	AtMYB30	Defense	293	3
tcwd	Bv5g112510_tcwd	5	31,754,754	31,740,788	C37 (MYB3R)	AtMYB3R1	Cell cycle	549	7
nmrg	Bv5g115970_nmrg	5	42,841,475	42,839,260	C4 (S1)	AtMYB30	Defense	334	3
oaxt	Bv5g116880_oaxt	5	44,348,080	44,350,042	C11	AtTDF1	Development	356	3
tfkh	Bv5g118200_tfkh	5	46,474,363	46,471,416	C38 (S25)	AtPGA37	Development	501	3
ahtj	Bv5g118320_ahtj	5	46,667,594	46,670,167	C38 (S25)	AtPGA37	Development	456	3
cfqe	Bv5g118940_cfqe	5	47,478,672	47,484,506				354	3
roao	Bv5g122000_roao	5	50,845,046	50,849,238	C9 (S2)	NtMYB1, AtMYB13	Defense	304	3
iogq	Bv5g122370_iogq	5	51,297,529	51,287,534	C13 (S7)	ZmP, AtPFG1	Metabolism	387	3
ijmc	Bv5g123335_ijmc	5	52,180,596	52,182,338	C33 (S20)	TaPIMP1, AtBOS1	Def., devel.	313	3
ohkk	Bv5ug126300_ohkk	5un	58,923,676	58,920,132	C39	AmPHAN, AtAS1	Development	369	1
knac	Bv5ug126380_knac	5un	59,366,406	59,371,048	C36 (S21)	AtLOF1, AtMYB52	Devel., metab.	354	3
qcwx	Bv5ug126530_qcwx	5un	60,109,952	60,111,819	C7 (S11)	AtMYB102	Defense	334	2
such	Bv6g128620_such	6	508,583	510,104	C34 (S22)	AtMYB44	Def., devel.	313	1
hwmt	Bv6g129790_hwmt	6	2,064,496	2,062,852	C12 (S14)	SlBLIND, AtRAX1	Development	321	3
oypc	Bv6g136060_oypc	6	9,418,772	9,413,559	C6 (S24)			326	3
usyi	Bv6g142590_usyi	6	24,870,820	24,874,245	C33 (S20)	TaPIMP1, AtBOS1	Def., devel.	275	3
qttn	Bv6g154730_qttn	6	58,819,129	58,822,997	C36 (S21)	AtLOF1, AtMYB52	Devel., metab.	461	3
zeqy	Bv6g155340_zeqy	6	59,766,073	59,770,527	C40	AtFLP	Differentiation	445	12
yejr	Bv7g162730_yejr	7	7,720,067	7,722,320	C12 (S14)	SlBLIND, AtRAX1	Development	380	3
ahzs	Bv7g172570_ahzs	7	37,491,045	37,494,304	C26	AtMYB26	Development	405	3
eztu	Bv7g172590_eztu	7	37,594,009	37,596,132	C26	AtMYB26	Development	417	3
qzms	Bv7g174540_qzms	7	40,066,216	40,070,163	C38 (S25)	AtPGA37	Development	481	3
ksge	Bv7g176420_ksge	7	42,070,277	42,071,595	C30	AtMYB59	Development	249	3
qzfy	Bv7ug180860_qzfy	7un	49,349,681	49,351,461	C31			264	3
dxny	Bv8g183050_dxny	8	719,286	716,855	C7 (S11)	AtMYB102	Defense	409	3
zguf	Bv8g183060_zguf	8	734,446	721,513	C7 (S11)	AtMYB102	Defense	396	3
jona	Bv8g199535_jona	8	37,703,170	37,704,769	C12 (S14)	SlBLIND, AtRAX1	Development	271	3
khqq	Bv8g200250_khqq	8	38,542,747	38,540,562	C12 (S14)	SlBLIND, AtRAX1	Development	368	3
gjwr	Bv9g216350_gjwr	9	33,882,588	33,884,593	C14 (S4)	AtMYB4, HvMYB5	Metabolism	276	2
krez	Bv9g225930_krez	9	44,634,979	44,633,906	C34 (S22)	AtMYB44	Def., devel.	253	1
ezhe	Bvg229250_ezhe	rnd0020	411,935	416,451	C28 (S18)	HvGAMYB, AtDUO1	Development	410	3
crae	Bvg229400_crae	rnd0039	39,535	40,725	C17 (S5)	AtTT2	Metabolism	282	3
entg	Bvg229850_entg	rnd0043	141,170	143,052	C33 (S20)	TaPIMP1, AtBOS1	Def., devel.	310	3
swwi	Bvg235150_swwi	rnd0157	176,581	179,181				191	3
oyjz	Bvg238960_oyjz	rnd0254	47,161	49,928	C3	AtLMI2	Development	239	3
dani	Bvg239075_dani	rnd0254	156,586	162,101	C24 (S16)	AtLAF1	Development	285	3
sjwa	Bvg239080_sjwa	rnd0254	178,734	182,264	C24 (S16)	AtLAF1	Development	278	3
pgya	Bvg243050_pgya	rnd0446	12,407	14,214				326	3

The genes are ordered by RefBeet pseudochromosomes, from north to south. The unique, immutable four-letter identifier (gene ID) is given in the first column. The modifiable, annotation-version-specific gene code describing the chromosomal assignment and position on pseudochromosomes is given in the second column. "un" indicates the assignment to a chromosome without position and "rnd" indicates scaffolds without chromosomal assignment. Clade classification and functional assignment is based on the NJ tree presented in Figure 3.

**Figure 1:**
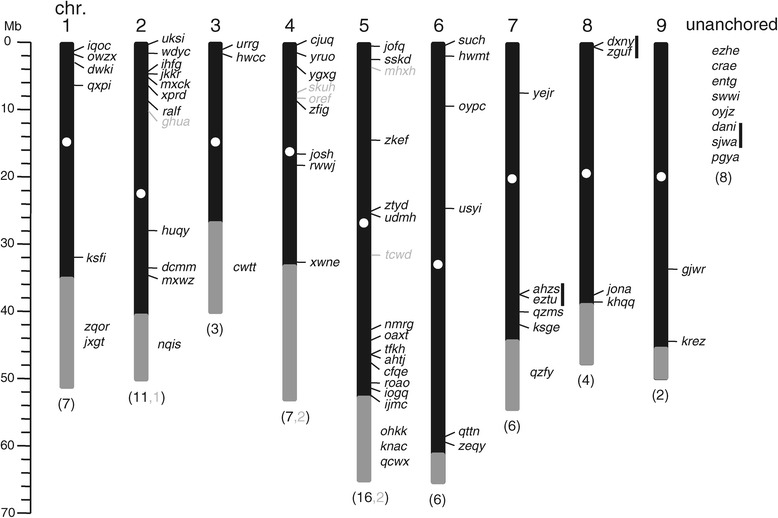
**Chromosomal distribution of** ***BvMYB*** **genes.** *R2R3-MYB* genes are present on all nine chromosomes in the *B. vulgaris* genome. Each broad vertical bar represents one chromosome drawn to scale. The black parts indicate concatenated scaffolds and grey parts mark scaffolds assigned to a chromosome without detailed position. The positions of centromeres (white dots) are roughly estimated from repeats distribution data. The chromosomal positions of the *MYB* genes (given in four-letter-ID) are indicated by horizontal lines. *R2R3-MYB* genes are given in black letters and other *MYB* genes are given in grey letters. The bracketed numbers below the chromosomes show the number of *MYB* genes on this chromosome. Eight *R2R3-MYB* genes could not be localised to a specific chromosome (unanchored). Vertical black lines indicate *R2R3-MYB* genes which are located in close proximity (sister gene pairs).

A keyword search in the NCBI database (http://www.ncbi.nlm.nih.gov/) revealed three previously annotated *B. vulgaris* MYB proteins from different sugar beet cultivars: AET43456 and AET43457, both corresponding to BvR2R3-MYB Bv_jkkr in this work and AEL12216, corresponding to Bv_nqis in this work. The identified 75 *BvMYB* genes, constituting approximately 0.27% of the 27,421 predicted protein-coding *B. vulgaris* genes and 5.9% of the 1271 putative *B. vulgaris* transcription factor genes [26], were subjected for further analyses. Similar to all other genes in the annotated *B. vulgaris* genome (RefBeet), a unique, immutable four-letter identifier (ID) was assigned to each *BvMYB* gene (Table 1). This immutable ID is part of the gene designator used in the *B. vulgaris* nomenclature system and should be stable, in contrast to the designator elements describing the chromosomal assignment and position on pseudochromosomes which may change when currently unassigned or unanchored scaffolds are integrated into the pseudochromosomes. Hereafter the four-letter-ID is used to name individual *BvMYB* genes and the deduced proteins. On the basis of RefBeet, 67 of the 75 *BvMYB* genes could be assigned to the nine chromosomes. On average, one *R2R3-MYB* gene was present every 10.5 Mb. The chromosomal distribution of *BvMYB* genes on the pseudochromosomes is shown in Figure 1 and revealed that *B. vulgaris MYB* genes were distributed throughout all chromosomes. Although each of the nine *B. vulgaris* chromosomes contained *MYB* genes, the distribution appeared to be uneven (Figure 1). The *BvMYB* gene density per chromosome was patchy, with only two *BvR2R3-MYB* genes present on chromosome 9, while 16 were found on chromosome 5. In general, the central sections of chromosomes including the centromeres and the pericentromere regions, lack *MYB* genes. Relatively high densities of *BvMYB* genes were observed at the chromosome ends, with highest densities observed at the top of chromosome 2 and at the bottom of chromosome 5 (Figure 1). This uneven distribution was previously observed for *Z. mays, G. max* and *M. x domestica R2R3-MYB* genes [15,22,25].

The total number of identified *MYB* genes was, compared to other plant species, low in *B. vulgaris*. Even if some MYB genes may have been missed due to gaps in the reference sequence, this does not adequately explain the small number. High numbers of *MYB* genes in a species are mainly attributed to ancestral whole genome duplication events as known for *A. thaliana, O. sativa, P. trichocarpa*, *G. max* and *M. x domestica* [27-31]. The absence of a recent lineage-specific whole genome duplication event in *B. vulgaris* [32] is further substantiated by the detection of only 70 *R2R3-MYB* genes, because a lack of this duplication event can easily explain the small number of *MYB* genes in this species. This interpretation is in accordance with the findings in cucumber, where the number of *R2R3-MYB* genes has been reported to be 55 [24].

We further determined physically linked sister *BvMYB* gene pairs along the nine chromosomes (Figure 1, marked with vertical black bars), which form clusters and may have evolved from local intrachromosomal duplication events that result in tandem arrangement of the duplicated gene. Three gene pairs have been identified: one on chromosome 7 consisting of the closely related genes *Bv_ahzu* and *Bv_eztu*, a second on the top of chromosome 8 with *Bv_dxny* and *Bv_zguf* and a third on an unlinked scaffold (0254.scaffold00675) constituted of *Bv_dani* and *Bv_sjwa*. The *BvMYB* genes of the two latter pairs were physically located near to each other without intervening annotated genes between. In total, about 5% (6 of 75) of *BvMYBs* were involved in tandem duplication, which is the same value as reported for MYB genes in soybean [15]. Moreover, an incomplete gene pair was observed on chromosome 2, where a solitary typically R2R3-MYB "third exon" containing sequences encoding a part of a R3 repeat and the C-terminal region was found about 18.7 kb downstream of *Bv_jkkr* showing 88% identity on cDNA- and 82% identity on deduced protein level to the third exon of the near *Bv_jkkr*.

Gene structure analysis revealed that most *BvR2R3-MYB* genes (53 of 70, 76%) follow the previously reported rule of having two introns and three exons, and display the highly conserved splicing arrangement that has also been reported for other plant species [15,22,24]. Eleven *BvR2R3-MYB* genes (16%) have one intron and two exons and four (6%) were one exon genes. Only two *BvR2R3-MYB* genes have more than three exons: *Bv_mxck* with four exons and *Bv_zeqy* with twelve exons (Table 1). The complex exon-intron structure of *Bv_zeqy* is known from its *A. thaliana* orthologs *AtMYB88* and *AtMYB124/FOUR LIPS (FLP)* containing ten and eleven exons, respectively, and more than the typically zero to two introns in the MYB domain coding sequences [18,19]. This supports their close evolutionary relationship, but also indicates the conservation of this intron pattern in evolution since the split of the Caryophyllales from the precursor of rosids and asterids.

### Sequence features of the MYB domains

**Figure 2:**
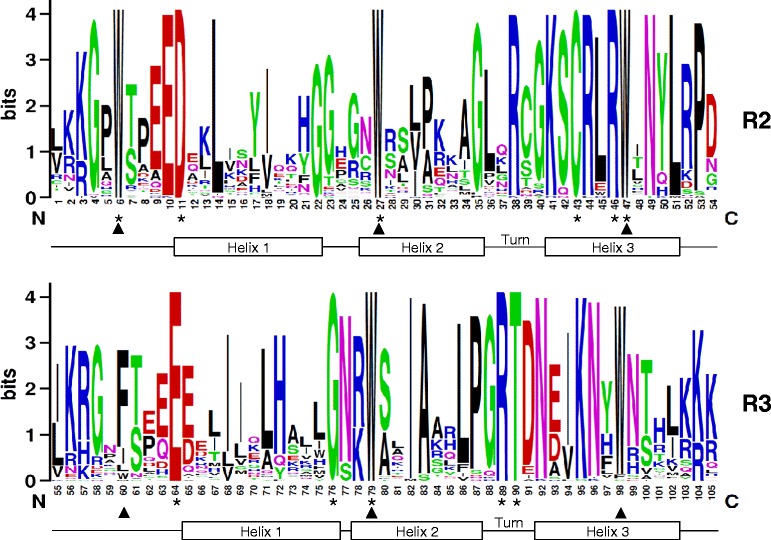
**Sequence conservation of the R2R3-MYB domain.** The R2 and R3 MYB repeats are highly conserved across all BvR2R3-MYB proteins. The logos base on alignments of all R2 and R3 MYB repeats of BvR2R3-MYBs. The overall height of each stack indicates the conservation of the sequence at the given position within the repeat, while the height of symbols within the stack indicates the relative frequency of each amino acid at that position. The asterisks indicate positions of the conserved amino acids that are identical among all 70 *B. vulgaris* R2R3-MYB proteins. Arrowheads indicate the typical, conserved tryptophan residues (W) in the MYB domain.

To investigate the R2R3-MYB domain sequence features, and the frequencies of the most prevalent amino acids at each position within each repeat of the *B. vulgaris* R2R3-MYB domain, sequence logos were produced through multiple alignment analysis (ClustalW) using the 70 deduced amino acid sequences of R2 and R3 repeats, respectively. In general, the two MYB repeats covered about 104 amino acid residues (including the linker region), with rare deletions or insertions (Additional file 1). As shown in Figure 2, the distribution of conserved amino acids among the *B. vulgaris* MYB domain was very similar to those of *A. thaliana, Z. mays, O. sativa, V. vinifera, P. trichocarpa, G. max* and *C. sativus.* The R2 and R3 MYB repeats of the *B. vulgaris* R2R3-MYB family contained characteristic amino acids, including the most prominent series of regularly spaced and highly conserved tryptophan (W) residues, which are known to play a key role in sequence-specific DNA binding, serving as landmarks of plant MYB proteins. As known from orthologs in other plant species, the first conserved tryptophan residue in the R3 repeat (position 60, W^60^) could be replaced by F or less frequently by isoleucine (I), leucine (L) or tyrosine (Y). Interestingly, the position 98 of the MYB repeat, which contains the last of the conserved tryptophan residues (W^98^), is not completely conserved in the *B. vulgaris* R2R3-MYBs (Figure 2, Additional file 1). A phenylalanine (F) residue, found in Bv_ralf and Bv_zeqy, has been reported very rarely at this position (e.g. in ZmMYB29) [22], but an atypical cysteine (C) at this important position, as found in Bv_jkkr, has not been described yet. This makes the R2R3-MYB protein Bv_jkkr interesting for further analyses in respect to DNA-binding and target sequence specificities.

In addition to the highly conserved tryptophan residues, we observed amino acid residues that are conserved in all *B. vulgaris* R2R3-MYBs: D^11^, C^43^, R^46^ in the R2 repeat and E^64^, G^76^, R^89^ and T^90^ in the R3 repeat. Further highly conserved residues of the *B. vulgaris* R2R3-MYB domains are: G^4^, E^10^, L^14^, G^35^, R^38^, K^41^, R^44^, N^49^, L^51^ and P^53^ in the R2 repeat and I^82^, A^83^, N^92^, K^95^ and N^96^ in the R3 repeat (Figure 2). These highly conserved amino acid residues are mainly located in the third helix and the turn of the helix-turn-helix (HTH) motif, which is in good accordance with the findings in other plant species.

### Phylogenetic analysis of the *B. vulgaris* MYB family

To explore the putative function of the predicted *B. vulgaris* MYBs, we assigned them to functional clades known from *A. thaliana*, which was chosen because most of our knowledge about plant *MYB* genes has been obtained from studies of this major plant model. As known from similar studies, most MYB proteins sharing similar functions cluster in the same phylogenetic clades, suggesting that most closely-related MYBs could recognise similar target genes and possess redundant, overlapping, and/or cooperative functions.

**Figure 3:**
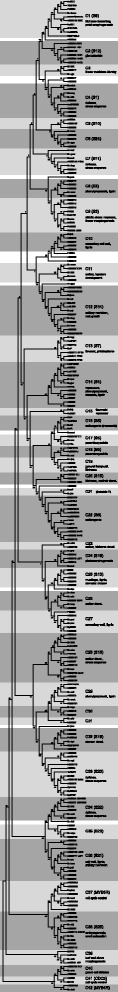
**Phylogenetic Neighbor Joining (NJ) tree (1000 bootstraps) with MYB proteins from** ***Beta vulgaris (Bv), Arabidopsis thaliana (At)*** **and landmark MYBs from other plant species built with MEGA5.2.** Clades (and Subgroups) are labeled with different alternating tones of grey background. Functional annotation of clade members are given. The numbers at the branches give bootstrap support values from 1000 replications.

We performed a phylogeny reconstruction of 75 BvMYBs, the complete *A. thaliana* MYB family (133 members, including 126 R2R3-MYB, five MYB3R, one MYB4R and one CDC5-MYB) and 51 well-characterised landmark R2R3-MYBs from other plant species, using the neighbour-joining (NJ) method (Figure 3) and the maximum parsimony (MP) method (Additional file 2) in MEGA5 [33]. With the exception of some inner nodes with low bootstrap support values, the phylogenetic trees derived from each method displayed very similar topologies. We took this as an indication of reliability of our clade- and subgroup designations. The phylogenetic tree topology allowed us to classify the analysed MYB proteins into 42 clades (C1 to C42) (Figure 3). In our classification of the *MYB* genes, we also considered the subgroup (S) categories from *A. thaliana* [2,17]. Our classification resulted in the same clusters as those presented in previous studies for grape and soybean [15,19]. As shown in Figure 3, 34 out of 42 clades were present both in *B. vulgaris* and *A. thaliana*. Thus, it is likely that the appearance of most *MYB* genes in these two species predates the branch-off of Caryophyllales before the separation of asterids and rosids [26].

We also observed species-specific clades and clades containing *B. vulgaris* or *A. thaliana* MYB proteins together with landmark MYBs from other species. It should be noted that we use the term "species-specific" in the context of the current set of species with sequenced genomes. Significantly more genome sequences would be required to resolve the presence and absence of genes or clades at the genus or family level. As indicated also from other studies, the observation of species-specific clades may be taken as a hint that *MYB* genes may have been acquired or been lost in a single species, during the following divergence from the most recent common ancestor. For example, members of the clade C2 (subgroup S12, with HIGH ALIPHATIC GLUCOSINOLATE1 (AtHAG1), HIGH INDOLIC GLUCOSINOLATE1 (AtHIG1), ALTERED TRYPTOPHAN REGULATION1 (AtATR1)) have been identified as glucosinolate biosynthesis regulators [34-37]. No BvMYBs were grouped within this clade, containing members which are predominantly present in plants of the glucosinolate compounds accumulating Brassicaceae family. A previous study indicated that this clade was derived from a duplication event before Arabidopsis diverged from Brassica [23]. Another MYB clade without *B. vulgaris* orthologs was C20 (S15), that include the landmark R2R3-MYBs AtMYB0/GLABRA1 and AtMYB66/WEREWOLF (Figure 3), which are known to be involved in epidermal cell development leading to the formation of trichomes and root hairs [38-40]. Similar observations have been made in the non-rosids maize and soybean [15,22], both not containing C20 (S15) orthologs, while the rosids grape and poplar do [19,20]. As *GLABRA1*-like *MYB* genes have been hypothesised to have been acquired in rosids after the rosid-asterids division [41,42], the absence of *BvMYBs* in this clade is consistent with this hypothesis, since Caryophyllales branched off before the separation of asterids and rosids. Trichome formation in *B. vulgaris* thus could be regulated by genes of the evolutionary older MIXTA clade C1 (S9) [41] whose members are also known to play a role in multicellular trichome formation [43]. Therefore, *Bv_dcmm* as the only sugar beet gene in this clade, is a candidate to encode a trichome development regulator. Further clades without BvMYBs are C5 (S10) containing the *A. thaliana* proteins AtMYB9, AtMYB107 with unknown functional assignment, C16 (S5) containing R2R3-MYB landmark anthocyanin regulators from monocots and C22 (S6) including the landmark R2R3-MYB factors PRODUCTION OF ANTHOCYANIN PIGMENT1 (AtPAP1), ANTHOCYANIN2 (PhAN2) and ROSEA1 (AmROSEA1), which are known to regulate anthocyanin biosynthesis in many species [44-47]. The lack of BvMYBs in the two latter clades fits to the observation, that plants of the genus *Beta* do not produce anthocyanin pigments [48]. The Caryophyllales is the single order in the plant kingdom that contains taxa that have replaced anthocyanins with chemically distinct but functionally identical red and yellow pigments - the indole-derived betalains, named from *Beta vulgaris*, from which betalains were first extracted [49]. Although betalains have functions analogous to those of anthocyanins as pigments, anthocyanins and betalains are mutually exclusive pigments in plants [50].

Three clades do not contain any *A. thaliana* MYB (Figure 3). Two of them contain landmark MYBs and *B. vulgaris* MYBs: C18 (S5) and C15, functionally assigned to proanthocyanidin regulation and repression of flavonoid biosynthesis, respectively. One clade contains only BvMYBs. Clade C21, constituted of the two BvMYBs (Bv_ralf and Bv_jkkr), was found close to the "anthocyanin" regulator representing clade C22 (S6) in the phylogenetic trees (Figure 3, Additional file 3). This clade could be described as a lineage-specific expansion in *B. vulgaris*, reflecting a species-specific adaptation. Two classical, linked beet pigment loci, RED (R) and YELLOW (Y) [51,52], are known to influence the production of betalains in beet. Recently, the R locus was shown to encode a cytochrome P450 (CYP76AD1, *Bv_ucyh* in KWS2320) [53]. It has been hypothesised that the betalain pathway may have co-opted the anthocyanin regulators because both pigment types are produced in a similar temporal and spatial pattern [53]. Thus, a R2R3-MYB-type transcriptional activator, homologues to the anthocyanin pigment regulators, is thought to control betalain biosynthesis through regulation of the biosynthetic enzymes R (CYP76AD1) and DODA (4,5-DOPA-dioxygenase) [53,54]. The two C21 *BvMYB* genes *Bv_jkkr* and *Bv_ralf* are both located on chromosome 2 close to the R locus, as indicated by the chromosome-based gene designations (*Bv2g027795_jkkr, Bv2g029890_ucyh, Bv2g030925_ralf*). As the R and Y loci are known to be linked at a genetically distance of about 7.5 cM [51,52], this makes the *R2R3-MYB* genes *Bv_ralf* and *Bv_jkkr* candidates for encoding the Y regulator. A closer inspection of the MYB domains of Bv_ralf and Bv_jkkr, and comparison to anthocyanin regulator MYBs, revealed some interesting features of C21 clade MYBs which could cause the separation of C21 and C22 MYBs (Figure 3). C21 MYBs do not contain the bHLH-binding consensus motif [D/E]Lx_2_[R/K]x_3_Lx_6_Lx_3_R [13] found in all bHLH-interacting R2R3-MYBs, suggesting that Bv_ralf and Bv_jkkr, in contrast to the C22 anthocyanin regulator MYBs, do not interact with bHLH proteins. A key amino acid residue in the R3 repeat, identified by Heppel *et al.* [55] in separating anthocyanin regulators (A^89^) from proanthocyanidin regulators (G^89^), is represented by isoleucine (I^89^) in C21 MYBs. Furthermore, one of the amino acid residues in the third helix of R2 repeat, known to be directly involved in DNA binding (position 44), differs from those found at this position in anthocyanin regulators (Additional file 1), maybe indicating a different target promoter specificity. In this direction also the atypical cysteine at the highly conserved position 98 in the MYB domain of Bv_jkkr may be important as discussed below.

A motif search in the BvMYB proteins for the above mentioned bHLH-interaction motif [13] identified seven BvMYBs containing this motif and thus putatively interacting with bHLH proteins. These seven BvMYBs were all functionally assigned to clades containing (potentially) known bHLH-interacting R2R3-MYBs: Bv_crae, Bv_swwi and Bv_dwki were assigned to the proanthocyanidin regulator clade C17 (S5), Bv_ihfg to clade C15 containing the negative flavonoid regulator FaMYB1 [56], Bv_cjuq to clade C19 containing the general phenylpropanoid pathway regulator VvMYB5a [57] and Bv_gjwr and Bv_iquc to the "repressors" clade C14 (S4).

Six *B. vulgaris* MYB proteins, Bv_uksi, Bv_josh, Bv_cfqe Bv_ijmc, Bv_swwi and Bv_pgya did not cluster in any of the identified clades or subgroups or showed ambiguous placements between the different phylogenetic trees, implying that these BvMYB proteins may have specialised roles that were acquired or expanded in *B. vulgaris* during the process of genome evolution.

Basically, higher numbers of AtMYBs than their BvMYB orthologs were found in most clades, indicating that they were duplicated after the divergence of the two lineages. For example, the phylogeny for C28 (S18) and C36 (S21) included only three BvMYBs and eight AtMYBs, or C25 (S13) with one BvMYB and four AtMYBs. By contrast, three BvMYBs and two AtMYB were found in clade C26. As mentioned above, the higher number of *MYB* genes in *A. thaliana* is presumably mainly due to an ancestral duplication of the entire genome and subsequent rearrangements, followed by gene loss and extensive local gene duplications taken place in the evolution of Arabidopsis [27].

### Expression profiles for *B. vulgaris MYB* genes in different developmental stages

In large transcription factor families functional redundancy is not unusual. Thus, a particular transcription factor has often to be studied and characterised in the context of the whole family. In this context, the gene expression pattern can provide important clues for gene function. We used genome-mapped global RNA-seq *Illumina* reads from the *B. vulgaris* reference genotype KWS2320 [26] to analyse the expression of the 75 *BvMYB* genes in different organs and developmental stages: seedlings, taproot (conical white fleshy root), young and old leaves, inflorescences and seeds. Filtered RNA-seq reads were aligned to the genome reference sequence and the number of mapped reads per annotated transcript were quantified and statistically compared across the analysed samples giving normalised RNA-seq read values which are given in Additional file 3. In common with other transcription factor genes, many of the *BvMYBs* exhibited low transcript abundance levels, as determined by the RNA-seq analysis.

**Figure 4:**
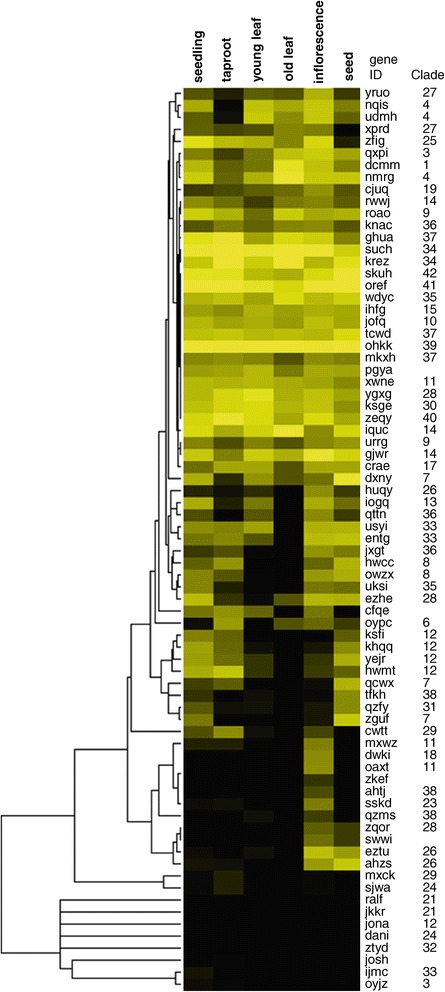
**Hierachical clustered heatmap showing the expression of** ***BvMYB*** **genes in different** ***B. vulgaris*** **(KWS2320) organs** ***.*** Normalised RNA-seq data was used to generate this heatmap. Expression values are indicated by intensity of yellow colour. Black indicates no detected expression. The hierachical clustering was performed using Cluster 3.0 software; for visualisation Java TreeView was used. Clade assignment of the BvMYBs is given at the right.

Our expression analysis revealed that *B. vulgaris* MYBs have a variety of expression patterns in different organs. We undertook hierarchical clustering of the profiles to identify similar transcript abundance patterns and generated an expression heatmap in order to visualise the different expression profiles of the *BvMYBs* (Figure 4). The highest number of *BvMYB* genes (66; corresponding to 88%) are expressed in inflorescences, followed by seedlings (62; 83%), taproot (58; 77%), seeds (55; 73%) and young leaves (51; 68%). The fewest *BvMYB* genes are expressed in old leaves (35; 47%). 70 *BvMYBs* (93%) are expressed in at least one of the analysed samples, although the transcript abundance of some genes was very low. 33 *BvMYB* genes (44%) were expressed in all samples analysed, which suggested that these *BvMYBs* play regulatory roles at multiple developmental stages. Five genes lacked expression information in any of the analysed samples, possibly indicating that these genes are expressed in other organs (e.g. root, stem), specific cells, at specific developmental stages, under special conditions or being pseudogenes. Some *BvMYB* genes are expressed in all analysed samples at similar levels (e.g. R2R3-MYB *Bv_ohkk* and MYB3R-type *Bv_tcwd*), while other show variance in transcript abundance with high levels in one or several organs and low (no) levels in others. For example, *Bv_hwcc, Bv_owzx* and *Bv_jxgt* are expressed in seedlings, taproot, inflorescence and seed, but not in leaves (neither young nor old leaves). Only four *BvMYB* genes, *Bv_oaxt, Bv_ahtj*, *Bv_zkef and Bv_ijmc*, three of them functionally assigned to *development*, show organ-specific expression and were only detected in inflorescences or seedlings. This result suggests that the corresponding BvMYB regulators are limited to discrete organs, tissues, cells or conditions.

Some clustered *BvMYB* genes (Figure 1), which are often considered as paralogs (created by a duplication event within the genome), showed similar expression profiles, while other clustered *BvMYB* genes did not. *Bv_ahzs* and *Bv_eztu* (both in the *development*-related clade 26), are mainly expressed in inflorescences and seeds with only low (or no) expression in the other analysed organs. The two clustered genes *Bv_dxny* and *Bv_zguf* (both in the *defence*-related clade 7) showed different expression profiles with *Bv_zguf* mainly expressed in seedlings and seeds and *Bv_dxny* further expressed in leaves. These results indicate, that *Bv_ahzs* and *Bv_eztu* could be functionally redundant genes, while *Bv_dxny* and *Bv_zguf* could be (partly) involved in various aspects of defence processes.

### Functional analysis of the *R2R3-MYB* gene *Bv_iogq*

In the context of functional analysis we selected *Bv_iogq* to start with. Bv_iogq is the only BvMYB in clade C13 (S7) clustering together with landmark MYBs having been implicated as flavonol- and phlobaphene-specific activators of flavonoid biosynthesis during plant development. This especially includes the *Z. mays* factor ZmP, controlling the accumulation of red phlobaphene pigments in pericarps [58], the *V. vinifera* flavonol regulator VvMYBF1 [59] and the *A. thaliana* PRODUCTION OF FLAVONOL GLYCOSIDES (PFG) family constituted of AtMYB12/AtPFG1, AtMYB11/AtPFG2 and AtMYB111/AtPFG3 [60]. AtMYB12 was shown to regulate CHALCONE SYNTHASE (CHS) expression by binding the MYB-recognition element MRE^*CHS*^ [61]. The three *A. thaliana* PFG-family factors were further found to have overlapping functions in flavonol pathway-specific gene regulation but to play organ-specific roles. Concordantly, *A. thaliana* seedlings of the *pfg* triple mutant *(myb11 myb12 myb111)* do not form flavonols under standard growth conditions, whereas the accumulation of other flavonoids is not affected [60].

We tested if *Bv_iogq* codes for a functional flavonol regulator. The *Bv_iogq* open reading frame (ORF) encodes a predicted protein with 387 amino acid residues, with a calculated molecular mass of 43.6 kD. Analysis of the deduced amino acid sequence revealed that Bv_iogq contains both motifs which have been described to be characteristic for flavonol regulators. The subgroup S7 motif (GRTxRSxMK) [17] was found to be present in the C-terminus of Bv_iogq with one amino acid substitution (GRTSRWAMQ), and the SG7-2 motif ([W/x][L/x]LS; [59]) was found at the very C-terminal end of Bv_iogq.

Since flavonol glycosides are known to accumulate in *B. vulgaris* leaves [62,63], and our expression data indicated *Bv_iogq* to be expressed in young leaves (Figure 4), this organ was chosen as source to clone the cDNA of the putative flavonol biosynthesis regulator Bv_iogq.

### Bv_iogq activates promoters of the flavonoid pathway structural genes

**Figure 5:**
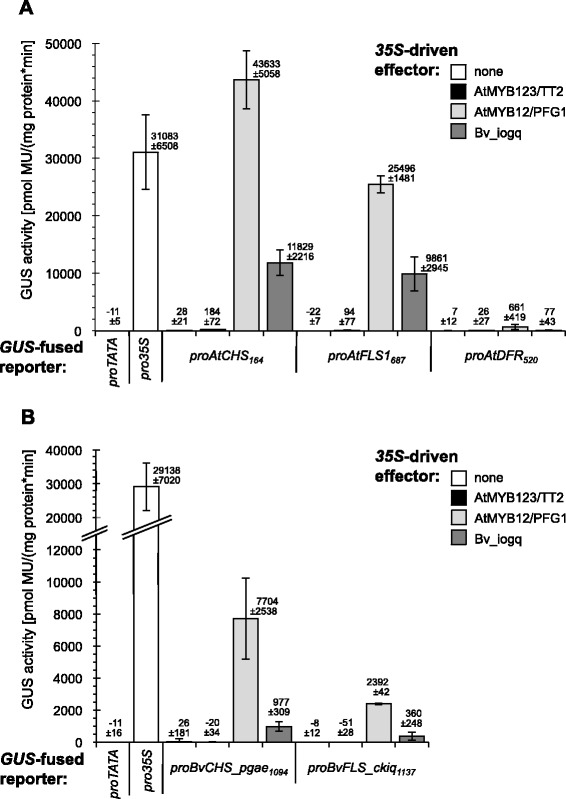
**Co-transfection analysis in At7 protoplasts indicate** ***in vivo*** **regulatory potential of Bv_iogq on** ***A. thaliana*** **and** ***B.vulgaris CHS*** **and** ***FLS*** **promoters.** Results from co-transfection experiments in *A. thaliana* protoplasts. Promoter fragments of the **(A)** *A. thaliana AtCHS, AtFLS1* and *AtDFR* genes, and **(B)** *B. vulgaris BvCHS_phae* and *BvFLS_ckiq* genes (reporters) were assayed for their responsiveness to the effectors AtMYB12/PFG1, AtMYB123/TT2 and Bv_iogq expressed under the control of the CaMV 35S promoter. Subscripted numbers indicate promoter fragment length. The figure shows mean GUS activity resulting from the influence of tested effector proteins on different reporters. Data from a set of four replicates are presented.

A transient *A. thaliana* At7 protoplast reporter gene assay system was used to analyse the activation potential of Bv_iogq on promoters of *A. thaliana* flavonoid biosynthesis enzymes. Figure 5A summarises the results and shows that Bv_iogq is able to activate the promoters of *AtCHS*, the initial enzyme in the flavonoid pathway, and *AtFLAVONOL SYNTHASE1 (AtFLS1)*, the enzyme catalysing the final step leading to the formation of flavonols. The promoter of the anthocyanidin branch gene *AtDIHYDROFLAVONOL REDUCTASE (AtDFR)*, whose product directly competes with FLS for the same substrate (dihydroflavonols), is not activated by Bv_iogq. In further experiments we tested the potential of Bv_iogq to activate the *B. vulgaris CHS_phae* and *FLS_ckiq* promoters in At7 protoplasts. The results of the transfection assays (Figure 5B) show that AtMYB12 activates both tested flavonoid *B. vulgaris* promoters, even though to a lower extent than the *A. thaliana* homologs. Furthermore, Bv_iogq is able to activate the promoters of *BvCHS_phae* and *BvFLS_ckiq* to some extend. We therefore claim Bv_iogq a functional flavonoid regulator, also in consideration of the heterologous assay system. These results revealed substantial functional similarities between the R2R3-MYB transcription factors AtMYB12 and Bv_iogq, as predicted by structural similarities (Figure 2). AtMYB12 was previously shown to activate a subset of flavonoid pathway genes without the need of a bHLH cofactor [61]. Bv_iogq also lacks the bHLH interaction motif in the R2R3 domain, thereby activating a subset of flavonoid pathway genes without the need of a cofactor (Figure 5), confirming that flavonol synthesis does not depend on bHLH cofactors in *A. thaliana* and *B. vulgaris*. The results of the transactivation assay suggest Bv_iogq to be a specific regulator of flavonol biosynthesis potentially regulating the early biosynthesis genes directing flavonoid precursors to flavonol formation in *B. vulgaris*.

### Complementation of the flavonol-deficient phenotype of *A. thaliana* multiple *pfg* mutant

**Figure 6:**
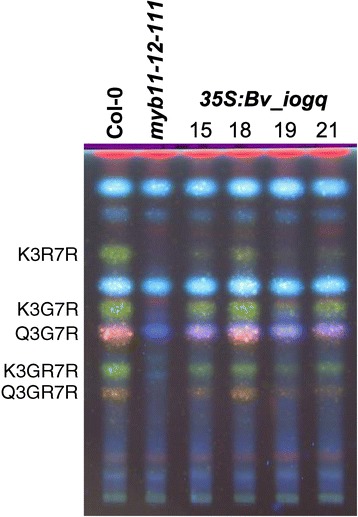
***35S:Bv_iogq*** **complements the flavonol-deficient** ***A. thaliana myb11-12-111*** **mutant.** HPTLC of methanolic extracts of T2 seedlings of independent transgenic *A. thaliana* lines (15, 18, 19, 21) containing T-DNA insertions with a *35S:Bv_iogq* construct in the flavonol-deficient *myb11-12-111* background. Flavonol glycosides were detected by DPBA staining and visualisation under UV illumination, indicating kaempferol derivatives (green) and quercetin derivatives (orange). K3R7R, kaempferol-3-*O*-rhamnoside-7-*O*-rhamnoside; K3G7R, kaempferol-3-*O*-glucoside-7-*O*-rhamnoside; Q3G7R, quercetin-3-*O*-glucoside-7-*O*-rhamnoside; K3GR7R, kaempferol-3-*O*- glucorhamnosid-7-*O*-rhamnoside; Q3GR7R, quercetin-3-*O*- glucorhamnosid-7-*O*-rhamnoside.

To confirm the function of Bv_iogq as a regulator of flavonol synthesis, the *Bv_iogq* ORF was expressed under the control of the constitutive cauliflower mosaic virus 35S promoter (*2x35S:Bv_iogq*) in the *A. thaliana myb11 myb12 myb111* triple mutant. Whereas seedlings of the mutant showed flavonol-deficient phenotype, several, independent stable transgenic *myb11 myb12 myb111*::2×35S:Bv_iogq lines showed flavonol accumulation, as visualised by diphenylboric acid-2-aminoethylester (DPBA) staining of high performance thin layer chromatography (HPTLC) separated flavonols (Figure 6). This result clearly shows that Bv_iogq is able to complement the *A. thaliana myb11 myb12 myb111* mutant deficient in flavonol synthesis. Therefore, Bv_iogq from *B. vulgaris* is a functional R2R3-MYB transcription factor involved in the regulation of flavonol biosynthesis. To indicate this function, we named *Bv_iogq* according to the *A. thaliana* ortholog: *BvMYB12 (BvPFG1)*.

## Conclusions

The genome-wide identification, chromosomal organisation, functional classification and expression analyses of *B. vulgaris MYB* genes provide an overall insight into this transcription factor-encoding gene family and their potential involvement in growth and development processes. This study provides the first step towards cloning and functional dissection to uncover the role of *MYB* genes in this economically and evolutionarily interesting species, as representative for the order Caryophyllales. The functional classification was successfully verified by experimental confirmation of the prediction that the *R2R3-MYB* gene *Bv_iogq* encodes a flavonol regulator, thus making BvMYB12 the first sugar beet R2R3-MYB with an experimentally proven function.

## Methods

### Database search for MYB protein coding genes in the *B. vulgaris* genome

An initial search for MYB protein coding genes in *B. vulgaris* was performed using a consensus R2R3-MYB DNA binding domain sequence [19] as protein query in TBLASTN [64] searches on the beet genome sequence (RefBeet, http://www.genomforschung.uni-bielefeld.de/en/projects/annobeet). To confirm the obtained amino acid sequences, the putative MYB sequences were manually analysed for the presence of an intact MYB domain. All *B. vulgaris* MYB proteins were inspected to ensure that the putative gene models contained two or more (multiple) MYB repeats, and that the gene models mapped to unique loci in the genome. Redundant sequences were discarded from the data set to obtain unique *BvMYB* genes. The identified *BvMYB* genes were matched with the automatically annotated *MYB* genes from the study of Dohm *et al*. ([26]). The open reading frames of the identified *BvMYB* have been verified by mapping, visualisation and manually inspection of combined RNA-seq data. Additionally, manually identified *BvMYB* genes received a unique immutable four-letter-ID and a gene code describing the position on pseudochromosomes or unanchored scaffolds. Multiple FASTA files with protein sequences used in this work, CDS and CDSi (CDS plus introns) sequences of *BvMYBs* are found as Additional files 4, 5 and 6.

### Sequence conservation analysis

To analyse the sequence features of the MYB domain of *B. vulgaris* R2R3-MYB proteins, the sequences of R2 and R3 MYB repeats of 70 BvR2R3-MYB proteins were aligned with ClustalOmega (1.2.0) (http://www.ebi.ac.uk/Tools/msa/clustalo/) using the default parameters. The sequence logos for R2 and R3 MYB repeats were produced from the multiple alignment files by the web based application *WebLogo* (http://weblogo.berkeley.edu/logo.cgi) [65] using default settings.

### Phylogenetic analyses

133 *A. thaliana* MYB protein sequences were obtained from TAIR (http://www.arabidopsis.org/). 51 well-known landmark plant R2R3-MYB protein sequences were collected from GenBank at the National Center for Biotechnology Information (NCBI) (http://www.ncbi.nlm.nih.gov/). We additionally considered atypical multiple MYB-repeat proteins from *A. thaliana* in the phylogenetic analysis to determine orthologs in the *B. vulgaris* genome: AtCDC5, AtMYB4R1 and five AtMYB3R. Phylogenetic trees were constructed from ClustalW aligned full-length MYB proteins (75 BvMYBs, 133 AtMYBs and 51 plant landmark MYBs) using MEGA5.2 [33] with full-length proteins and default settings. Two statistical methods were used for tree generation: (i) neighbor-joining (NJ) method with poison correction and bootstrap analysis with 1000 replicates, (ii) maximum parsimony (MP) method with 1000 bootstrap replications. Classification of the *B. vulgaris* MYBs was performed according to their phylogenetic relationships with their corresponding *A. thaliana* and landmark MYB proteins.

### Expression analysis from RNA-sequencing data

RNA-seq raw data from inflorescences, taproots, old leaves, seeds and seedlings of the *B. vulgaris* double haploid reference line KWS2320 have been produced in the work of Dohm *et al.* [26] and are deposited at the NCBI Short Read Archive (SRA) under the accession numbers SRX287608-SRX287615. RNA-seq raw data from RNA isolated from young leaves (third and forth leaf without midrip) have been generated by paired-end sequencing (2× 101 bases) on an *Illumina HiSeq1500* instrument and are deposited together with a detailed documentation at the SRA under the accession number SRX647324. Raw RNA-seq reads were trimmed using Trimmomatic-0.22 [66] and the options ILLUMINACLIP, LEADING:3, TRAILING:3, SLIDINGWINDOW:4:15 as well as MINLEN:36. For Tophat [67] mappings versus the RefBeet sequence, only trimmed paired reads generated by Trimmomatic-0.22 were used (Additional file 7). For mapping of long read sets (>55 bases read length) and short read sets (≤55 bases read length) the mate inner distance was set to 175 and 150, respectively and the option *solexa1.3-quals* was switched on. Mapping results are displayed in Additional file 7. Resulting BAM files were quality filtered according a minimum mapping quality of five and converted using SAMtools [68]. SAM files were sorted afterwards (Unix command sort -s -k 1,1). Reads per gene were counted based on manually corrected gene structures stored in gff3 format and the sorted SAM files by htseq-count (option t = CDS) that is part of HTSeq version 0.5.4 (http://www-huber.embl.de/users/anders/HTSeq/doc/overview.html). Single count tables for each organ created by HTSeq were joined using a customised Perl script and subsequently imported in R by means of the DESeq package [69]. Data design, sample assignment and normalisation were performed as described previously [69]. The correction factors of different data sets were computed as 4.02, 1.25, 3.53, 0.11, 1.47, 0.41 for seedling, taproot, young leaf, old leaf, inflorescence and seed, respectively.

### Hierachical clustering and heatmap visualisation of RNA-seq data

Centroid-linkage hierachical clustering of log2 transformed normalised RNA-seq data was performed using Cluster 3.0 software [70] (http://bonsai.hgc.jp/~mdehoon/software/cluster/software.htm). The hierachical clustering results were visualised using Java TreeView [71] (http://jtreeview.sourceforge.net).

### Plant material and growth conditions

*B. vulgaris* KWS2320 seeds were surface-sterilised with 70% ethanol and sown on sterile, wet cotton wool in a covered plastic beaker. Seedlings were grown for one week at 22°C giving 16 hours light per day. All other plant material was harvested from greenhouse/soil-grown *B. vulgaris* plants.

### RNA isolation and cDNA synthesis

RNA was isolated from 50 mg plant material using the *NucleoSpin*®*RNA Plant* kit (*Macherey-Nagel*) according to the manufacturers instruction. cDNA was generated from 1 μg total RNA as described previously [60].

### cDNA cloning

A cDNA fragment corresponding to the full-length ORF of *Bv_iogq* (1164 bp) was obtained by PCR using attB recombination sites containing sense RS1050 (5'-attB1-ccATGGGGAGAGCACCGTGTTGCGAAAAG-3') and antisense RS1051 (5'-attB2-gTTATGAAAGTAGCCAATCAAGCATAGC-3') primers with Phusion® polymerase (*New England BioLabs*) on cDNAs prepared from *B. vulgaris* KWS2320 young leaves as template. The resulting PCR product was introduced into the GATEWAY® vector pDONRzeo using BP clonase (*Invitrogen*), giving a *Bv_iogq* entry clone (RSt810). The *Bv_iogq* cDNA sequence was submitted to EMBL/GenBank; accession number KJ707238.

### Complementation experiments with *Bv_iogq*

The *Bv_iogq* entry clone was recombined by LR-reaction (*Invitrogen*) into the binary destination vector pLEELA [72] giving a *2×35S:Bv_iogq* construct (RSt815). The resulting plasmid was used in a complementation experiment transforming the flavonol-deficient *A. thaliana* triple mutant *myb11 myb12 myb111* (NASC N9815) via *Agrobacterium tumefaciens* (GV3101::pM90RK) [73] according to the floral dip protocol. Flavonol-containing methanolic extracts of T2 seedlings of different transgenic lines were analysed by high performance thin layer chromatography (HPTLC) and diphenylboric acid 2-aminoethyl ester (DPBA)-staining as described elsewhere [60].

### Transient protoplast assays

Growth of *A. thaliana* At7 cell culture, protoplast isolation and transfection experiments for the detection of transient expression were performed as described by Mehrtens *et al.* [61]. In the co-transfection experiments, a total of 25 μg of pre-mixed plasmid DNA was transfected, consisting of 10 μg of reporter plasmid (transcriptional GUS fusion), 10 μg of effector plasmid and 5 μg of the luciferase (LUC) transfection control (*4xproUBI:LUC*) [74]. Protoplasts were incubated for 20 h at 26°C in the dark before LUC and GUS enzyme activities were measured. Specific GUS activity is given in pmol 4-methylumbelliferone (MU) per mg protein per min. The error bars indicate the standard deviation of four GUS values determined for the respective reporter/effector combination. The *35S:Bv_iogq* effector plasmid (RSt816) was generated by LR-reaction *(Invitrogen)* of the *Bv_iogq* Entry clone (RSt810) with the destination vector pBTdest [75]. A 1064 bp promoter fragment of *proBvCHS_phae* was obtained by PCR on genomic *B. vulgaris* KWS2320 DNA using attB recombination sites containing primers BP60 (5'-attB1-CCACAAGAACATCTTGTAAGAGC-3') and BP55 (5'-attB2-CAACTCGTGAGTATGAAGAAATATG-3'). The 1137 bp promoter fragment of *proBvFLS_ckiq* was gained with the attB primers RS1128 (5'-attB1-TATACCTTGTAATATCACTTAAATCT-3') and RS1129 (5'-attB2-CCATTGAATATCAACTCAGGATTTTC-3'). The resulting PCR products were introduced via BP and LR reactions into the GATEWAY® destination vector pDISCO [76] giving the *proBvCHS_phae-GUS* (DH33) and *proBvFLS_ckiq-GUS* (RSt830) reporter constructs. The other effector- and reporter constructs have been described elsewere [13,61,77].

### Availability of supporting data

Phylogenetic data (trees and the data used to generate them) have been deposited in TreeBASE respository and is available under the URL http://purl.org/phylo/treebase/phylows/study/TB2:S16360.

RNA-seq data from inflorescences, taproots, young leaves, old leaves, seeds and seedlings of the *B. vulgaris* line KWS2320 are deposited at the NCBI Short Read Archive (SRA; http://www.ncbi.nlm.nih.gov/sra/) under the accession numbers SRX287608-SRX287615 and SRX647324.

## Additional files

Additional file 1:
**Multiple alignment of MYB domains of 70** ***B. vulgaris*** **R2R3-MYB proteins. **ClustalW amino acid sequence alignment. The black arrowheads at the top indicate the conserved, regulary spaced tryptophan (W) or phenylalanine (F) residues. Position numbers at the top correspond to those given in Figure 2. Additional file 2:
**Phylogenetic Maximal Parsimony (MP) tree (1000 bootstraps) with MYB proteins from** ***B. vulgaris (Bv), A. thaliana (At)*** **and landmark MYBs from other plants built with MEGA5.2.** Bootstrap measures are given at the branches. Clades (and Subgroups) are labelled and indicated by different shades of grey. Additional file 3:
**Expression of** ***BvMYB*** **genes in organs of the** ***B. vulgaris*** **reference line KWS2320, deduced from RNA-seq data from the study of Dohm** ***et al.*** **([** 26 **]).** Reads per gene were counted based on *BvMYB* gene structures. Single count tables for each organ were transformed to normalised data according to the method of Anders and Huber (2010), using correction factors for the different data sets which have been computed as 4.02, 1.25, 3.53, 0.11, 1.47, 0.41 for seedlings, taproot, young leaf, old leaf, inflorescence, seed, respectively. Additional file 4:
**Multiple FASTA file with protein sequences used in this work.**
Additional file 5:
**Multiple FASTA file with CDS sequences of** ***BvMYBs*** **.**
Additional file 6:
**Multiple FASTA file with CDSi (CDS plus introns) sequences of** ***BvMYBs*** **.**
Additional file 7:
**Trimming and mapping of RNA-seq reads. (A)** Trimming of RNA-seq raw reads using *Trimmomatic*. SRA: Sequence read archive. **(B)** Mapping of RNA-seq reads to RefBeet after applying *Tophat*.
