# Supplementary material for: Genome-wide identification and characterisation of R2R3-MYB genes in sugar beet (Beta vulgaris)
Source: BMC Plant Biol. 2014 Sep 25;14:249. doi: 10.1186/s12870-014-0249-8 (PMC4180131; doi:10.1186/s12870-014-0249-8)
Supplement: Additional file 1: — Multiple alignment of MYB domains of 70 B. vulgaris R2R3-MYB proteins. ClustalW amino acid sequence alignment. The black arrowheads at the top indicate the conserved, regulary spaced tryptophan (W) or phenylalanine (F) residues. Position numbers at the top correspond to those given in Figure 2. [file 12870_2014_249_MOESM1_ESM.doc]

10 20 30 40 50 60 70 80 90 100

• • • • • • • • • •

⯆ ⯆ ⯆ ⯆ ⯆ ⯆

Bv_qttn CARGHWRPAEDSKLKELV-AIYGP-----QNWNLIAEKLE---GRSGKSCRLRWFNQLDPRINRR-AFTEEEEERLMQAHRVYGNKWAMIARLFPGRTDNAVKNHWHVIMARK 103

Bv_jxgt CPRGHWRPAEDEKLRKLV-EQYGP-----QNWNSIAEKLQ---GRSGKSCRLRWFNQLDPRINRK-PFTEEEEERLLTAHRIHGNKWALISRLFPGRTDNAVKNHWHVIMARM 103

Bv_knac CSRGHWRPAEDAKLKQLV-LQFGP-----HNWNLIAEKLD---GRSGKSCRLRWFNQLDPRINKR-PFTEEEEERLKTAHKMYGNKWAVIARLFPGRTDNAVKNHWHVMKARE 103

Bv_krez RIKGPWSPEEDELLQKLV-QKHGA-----RNWSVISKSIP---GRSGKSCRLRWCNQLSPEVEHR-PFSTEEDEIIVRAHAQHGNKWATISRLLNGRTDNAIKNHWNSTLKRK 103

Bv_such RIKGPWSPEEDEALTRLV-QKHGP-----RNWSLISKSIP---GRSGKSCRLRWCNQLSPQVEHR-AFTPEEDDTIIRAHARFGNKWATIARLLSGRTDNAIKNHWNSTLKRK 103

Bv_uksi RVKGSWTPQEDATLIKLV-DQHGP-----RNWTLISEGIP---GRSGKSCRLRWCNQLSPDVQHR-PFSPSEDEIIIQAHKAHGNRWAVIAKLLPGRTDNAIKNHWNSTLRRK 103

Bv_wdyc RVRGPWSPEEDAILSELV-SKFGA-----RNWSLIARGIP---GRSGKSCRLRWCNQLDPCVKRK-PFSEEEDQIIISAHAIHGNKWACIAKLLPGRTDNAIKNHWNSTLRRR 103

Bv_tfkh CIKGQWTDKEDSLLKKLV-DQHGD-----RKWALIAEKMV---GRAGKQCRERWHNHLRPDIKKD-AWNEEEERILVAAHEQFGNRWAEIAKKLPRRTENSIKNHWNATKRRQ 103

Bv_qzms CIKGQWTIEEDRLLKLLV-KTYGV-----RKWALIAEKMV---GRAGKQCRERWHNHLRANIKKN-AWTEEEERILVAAHERFGNRWAEIAKLIDGRTENAIKNHWNATKRRQ 103

Bv_ahtj VVKGQWTIEEDRLLIQLV-ERYGV-----RKWSQIAQMLN---GRIGKQCRERWHNHLRPDIKKD-LWNEEEDRILIKAHAEIGNKWAEIAKKLPGRTENSIKNHWNATKRRQ 103

Bv_ahzs VKRGLWSPEEDEKLINYI-TTYGH-----GCWSSVPTLA--GLQRCGKSCRLRWINYLRPDLKRG-SFSPQEAALIIDLHRILGNRWSQIAKFLPGRTDNEVKNFWNSSIKKK 104

Bv_eztu VKRGLWSPEEDEKLISYI-TTYGH-----GCWSSVPRLA--GLQRCGKSCRLRWINYLRPDLKRG-SFSPQEATLIIDLHRILGNRWSQIAKYLPGRTDNEVKNFWNSSIKKK 104

Bv_huqy VKRGLWSPEEDEKLVSHI-TIHGH-----SSWSSVPKLA--GLQRCGKSCRLRWINYLRPDLKRG-SFTEEEERSIIEVHRILGNRWAQIAKHLPGRTDNEVKNFWNSCIKKK 104

Bv_zfig LRKGLWSPEEDEKLLRHI-TQYGH-----GCWSSVPKLA--GLQRCGKSCRLRWINYLRPDLKRG-AFSQEEENLIVELHAVLGNKWSQIAAQLPGRTDNEIKNLWNSCLKKK 104

Bv_cfqe LKKGLWSPEEDEKLYNHI-TRFGV-----GCWSSVPKLA--GLQRCGKSCRLRWINYLRPDLKRG-MFSQEEEGLIINLHQILGNRWAQIASQLPGRTDNEIKNFWNSCLKKK 104

Bv_sskd VKRGLWSPEEDEKLIRYI-TTHGY-----GCWSEVPEKA--GLQRCGKSCRLRWINYLRPDIRRG-RFTPEEEKLIISLHEVVGNRWAHIASHLPGRTDNEIKNYWNSWIKKK 104

Bv_sjwa HKKGLWSPEEDQRLKDYI-LQYGI-----SCWSSVPLIA--GLQRNGKSCRLRWINYLRPGLKRG-MFSTQEEKLILTLHQTLGNKWSQIAKHLPGRTDNEIKNYWHSYLKKR 104

Bv_dani HRKGLWSPEEDQNLRNYI-FQHGH-----GCWSSVPINA--GLQRSGKSCRLRWINYLRPGLKRG-TLSPQEEDTILNLHCALGNRWSQIALHLPGRTDNEIKNFWHSYLKKK 104

Bv_xprd LRKGLWSPEEDEKLMNYM-LRNGH-----GCWSDIARNS--GLQRCGKSCRLRWINYLRPDLKRG-AFSPQEEHLILHFHSLLGNRWSQIAARLPGRTDNEIKNFWNSTIKKR 104

Bv_yruo LRKGLWSPEEDDKLMNYM-LTNGQ-----GCWSDVARNA--GLLRCGKSCRLRWINYLRPDLKRG-AFSPQEEDLIIHLHSLLGNRWSQIAARLPGRTDNEIKNFWNSTVKKR 104

Bv_dxny LKKGPWTPEEDQKLLDYI-QKHGY-----GNWRTLPKNA--GLQRCGKSCRLRWTNYLRPDIKRG-RFSFEEEETIIQLHSILGNKWSAIAARLPGRTDNEIKNYWNTHIRKR 104

Bv_zguf LKKGPWLPEEDQKLVDYI-QKHGP-----GNWRTLPKKA--GLERCGKSCRLRWTNYLRPDIKRG-RFTFEEEEAIIQLHSVLGNKWSAIAAKLPGRTDNEIKNYWNTHIRKR 104

Bv_oypc LKKGPWTPEEDQKLVKFI-QKHGH-----GSWRALPKLA--GLNRCGKSCRLRWTNYLRPDIKRG-KFTPEEEQTILNLHAILGNKWSAIANHLPGRTDNEIKNFWNTHLKKK 104

Bv_hwcc LKKGPWTPEEDKQLTDYI-QRHGH-----GSWRALPKLA--GLNRCGKSCRLRWTNYLRPDIKRG-KFTDEEQQTIINLHSVLGNKWSAIASHLPGRTDNEIKNLWNTHLKKK 104

Bv_qcwx LKKGPWTPEEDEKLVSYI-QNHGH-----GSWRALPRAA--GLNRCGKSCRLRWTNYLRPDIKRG-RFTEEEEDMIIKLHSVLGNKWSRIAAHLPGRTDNEIKNYWNTHLRKK 104

Bv_zkef LKKGPWTPDEDEKLVSHI-QNHSH-----GSWRALAKAA--GLSRCGKSCRLRWHNYLRPDIKRG-QFSEEEDNLIIQLHSVLGNKWSQIAAHLPGRTDNDIKNYWNTRIKKK 104

Bv_nqis VKKGPWTPEEDILLVSYI-QEHGP-----GNWRAVPTNT--GLLRCSKSCRLRWTNYLRPGIKRG-NFTDQEEKMIIHLQALLGNRWAAIASYLPQRTDNDIKNYWNTHLKKK 104

Bv_nmrg VKKGPWTPEEDIILVSYI-QEHGP-----GNWRAVPTNT--GLRRCSKSCRLRWTNYLRPGIKRG-NFTEQEEKMIIHLQALLGNRWAAIASYLPQRTDNDIKNYWNTHLKKK 104

Bv_udmh IKKGPWTPEEDIILVSYI-QEHGP-----GNWRSVPTNTV-GLQRCSKSCRLRWTNYLRPGIKRG-NFTPHEEGMIIHLQALLGNKWAAIASYLPQRTDNDIKNYWNTHLKKK 105

Bv_dcmm LKKGPWTPEEDQKLLAYI-EEHGH-----GNWRALPTKA--GLQRCGKSCRLRWTNYLRPDIKRG-KFSLQEEQTIIQLHALLGNRWSAIATHLPKRTDNEIKNYWNTHLKKR 104

Bv_oyjz FKRGPWTPEEDQKLLHFIRNNNGHP----GNWKSLPQVA--GISRCGKSCRLRWTNYLRPDIRRG-PFSEEENETVIQLHGLLGNRWAAIAARLPGRTDNDVKNHWNSHLRKR 106

Bv_qxpi LKKGPWTTEEDELLVKYI-NQHGH-----GNWRSLPKNA--GLLRCGKSCRLRWTNYLRPDIKRG-PFTPEEEKLVIQLHAILGNRWAAIAAQLPGRTDNEIKNLWNTHLRKR 104

Bv_roao LKKGPWTPEEDQLLINYI-QINGH-----SNWRALPKQA--GLLRCGKSCRLRWTNYLRPDIKRG-NFTQEEEETIIKLHEMLGNRWSAIAAKLPGRTDNEIKNVWHTHLKKR 104

Bv_urrg LKKGPWTPEEDQILISFI-QQHGH-----SNWRALPKQS--GLLRCGKSCRLRWINYLRPDIKRG-NFSKEEEDTIIQLHEMMGNRWSAIAARLPGRTDNEIKNVWHTHLKKR 104

Bv_josh LKKGPWTPEEDQTLVSYI-QQFGH-----SNWRALPKQA--GLLRCGKSCRLRWTNYLRPDIKRG-NFSLEEEEIIIKMHQIVGNRWSTIATRLPGRTDNEIKNFWNINLKKK 104

Bv_owzx VKKGPWSQAEDYKLISFI-RKHGH-----NNWRALPKLA--GLARCGKSCRLRWVNYLRPDLKRG-NFTLQEEESIIKLHEMLGNKWSKIASQFPGRTDNEIKNVWNTHLKKR 104

Bv_jofq VKKGPWTAEEDKKLINFI-LTNGQ-----CCWRAVPKLA--GLRRCGKSCRLRWTNYLRPDLKRG-LLSDSEEQLVIDLHARLGNRWSKIAARLPGRTDNEIKNHWNTHIKKK 104

Bv_iquc TNKGAWTKDEDQRLIDYI-RLHGE-----GCWRSLPKAA--GLLRCGKSCRLRWINYLRPDLKRG-NFTEEEDELIIKLHSLLGNKWSLIAGRLPGRTDNEIKNYWNTHIKRK 104

Bv_gjwr TNKGAWTKEEDDRLIAYI-KAHGE-----GCWRSLPKAA--GLLRCGKSCRLRWINYLRPDLKRG-NFTEEEDELIIKLHSLLGNKWSLIAGRLPGRTDNEIKNYWNTHIKRK 104

Bv_rwwj TNKGAWTKEEDDRLVNYI-KSHGE-----GCWRSLPKAA--GLQRCGKSCRLRWINYLRPDLKRG-NFTEEEDELIINLHSLLGNKWSLIAAKLPGRTDNEIKNYWNTHIKRK 104

Bv_ihfg RNKGAWSKQEDQKLVDYI-KLHGE-----GCWHSLPKAA--GLDRCGKSCRLRWINYLKPDVKRG-NFAQDEEDLIIKLHALLGNRWSLIAGRLPGRTDNEVKNYWNSHIRKK 104

Bv_iogq LKRGRWTDEEDQKLMKYI-EENGE-----GSWRSMPKR----LLRCGKSCRLRWINYLRSDLRRG-NITPEEEEIIIKLHATMGNRWSMIAAQLPGRTDNEIKNYWNSHLSRK 102

Bv_crae LNKGAWTAMEDKILIDYI-KTHGE-----GRWRNLPKRA--GLKRCGKSCRLRWLNYLRPDIKRG-NITPDEEELIIRLHKLLGNRWSLIAGRLPGRTDNEIKNYWNTNLSKR 104

Bv_dwki LHRGPWTAREDVLLIKYI-EAHGE-----GQWRSLPKKA--GLLRCGKSCRLRWMNYLRPDIKRG-NITPDEDDLIIRLHGLLGNRWSLIAGRLPGRTDNEIKNYWNTHLSKK 104

Bv_swwi VNKGAWTAEEDTKLSDYI-QKYGA-----KQWKTVALKA--GVNRCGKSCRLRWLNYLRPNIKRG-NISDEEEDLIRRLHKLLGNRWSLIAGRLPGRTDNEIKNYWNSHLSKK 104

Bv_cjuq MKKGPWTAEEDELLRSYI-LTHQNQNGKVGGWRTVPQRA--GLLRCGKSCRLRWMNYLRPNLRRG-LFSSDEEDLILRLHRLLGNRWALIAGRIPGRTDNEIKNYWNTHLSKK 109

Bv_mxwz VKKGLWTPEEDAKILAYV-SNHGV-----GNWTQVPKKA--GLNRCGKSCRLRWTNYLRPDLKHE-EFTEQEEQLIIQYHAAIGSRWSLIAKHLPGRTDNDVKNYWNTKLKKK 104

Bv_oaxt VRKGAWVEEKDATNFAYL-PKQGG-----ANWSSVPRKT--GTKRCGKNCRLRWNNYMRPDLSHE-GFTQQEEERIIKLHAAVGSRWGLIAHQLPGRTENDIKIHWNTKLRKK 104

Bv_xwne VKRGQWTPEEDNKLSSYI-AQHGT-----RNWRLIPKNA--GLQRCGKSCRLRWTNYLRPDLKHG-QFSQPEEETIMKLHAVVGNRWSLIAAQLPGRTDNDVKNHWNTKLKKK 104

Bv_khqq VKKGPWSPEEDATLKAYI-EKYGTG----GNWIALPQKI--GLKRCGKSCRLRWLNYLRPNIKHG-GFTEEEDNIILSLYISIGSRWSIIAAQLPGRTDNDIKNYWNTRLKKK 105

Bv_hwmt VKKGPWSPEEDAKLKSYI-EAKGTG----GNWIALPQKI--GLKRCGKSCRLRWLNYLRPNIKHG-GFTEEEENIICSLYVSIGSRWSIIAAQLPGRTDNDIKNYWNTRLKKK 105

Bv_ksfi VKRGPWAPEEDAKLKSYI-EKHGTA----GNWIALPQKI--GLKRCGKSCRLRWLNYLRPNIKHG-KFSEEEDKVIISLYVSIGSRWSIIAAQLPGRTDNDIKNYWNTRLKKK 105

Bv_yejr VKKGPWSPEEDAKLKQYI-EKFGTG----GNWISLPQKA--GLKRCGKSCRLRWLNYLRPNIKHG-EFSDEEDRIICSLYASIGSRWSIIAAQLPGRTDNDIKNYWNTKLKKK 105

Bv_pgya VKRGPWSPEEDATLKNYL-HKHGTG----GNWISLPQRA--GLKRCGKSCRLRWLNYLRPDIKHG-SFTEEEDNIIISLFYKMGSRWSVIAANLPGRTDNDVKNHWNTKLKKK 105

Bv_jona VKKGPWSNEEDALLINFF-EKYGNG----GNWISLPKRA--GLNRCGKSCRLRWLNYLKPGIKHG-DFTEKEDYVIYTLYNSIGSRWSIIASHLQGRTDNDVKNYWNTKLKRK 105

Bv_jkkr VVKGSWSDEEDDLLRKCI-QKYGE-----GNWKRVPERA--GLNRCRKSCRWRWLNYLKPSIKRG-HFNEEEVKFIIQQHKLIGNRWSLIAAKLPGRTINDVKNYCDTHLYKK 104

Bv_ralf VKKGSWSDEEDELLCKCI-EKYGE-----GNWKRISERA--GLNRDRKSCRWRWLNYLKPNIKRG-PFGEDEIEFITQQHKLHGNRWSLIASRLPGRTINDVKNYFNTHIYKK 104

Bv_entg LRRGPWTVEEDLSLINYI-ATHGE-----GRWNSLARCA--GLKRTGKSCRLRWLNYLRPDVRRG-NITLEEQLLILELHSRWGNRWSKIAQYLPGRTDNEIKNYWRTRVQKH 104

Bv_ztyd VRKGPWTMEEDLILINYI-ANHGE-----GVWNSLARSA--GLKRTGKSCRLRWLNYLRPDVRRG-NITAEEELLIMELHAKWGNRWSKIAKHLPGRTDNEIKNYWRTRIQKH 104

Bv_usyi LRRGPWTLEEDTSLIHYI-HCHGE-----GRWNLLAKSA--GLKRTGKSCRLRWLNYLKPDIKRG-NLSPQEQLMILELHSKWGNRWSKIAAHLPGRTDNEIKNYWRTRVQKQ 104

Bv_ijmc LRKGPWTPQEDSKLTSSI-VIHGE-----GRWNALARLA--GLRRSGKSCRLRWLNYLRPDLRRG-PFTLQEQLLVFQLYFRWGNRWSKIAENLPGRTDNEIKNYWRTKVQKL 104

Bv_mxck WRKGPWTPEEDKLLVEYV-NLHGE-----GRWSSVARCA--GLNRSGKSCRLRWVNYLRPGLKRG-QITPQEEGIIIELHAIWGNKWSTIARYLPGRTDNEIKNYWRTHFKKK 104

Bv_cwtt WRKGPWTAEEDKLLIEYV-NLQGE-----GRWNSVARLT--GLRRNGKSCRLRWVNYLRPDLKRG-QITPHEESIILELHAKWGNRWSTIARSLPGRTDNEIKNYWRTHFKKK 104

Bv_qzfy LRKGPWYEEEDERLKAFV-AVLGA-----RRWDSVARIS--GLRRSGKSCRLRWLNYLRPFLKHG-PITPEEEHVIVKLHERWGNKWAKIARRLPGRTDNEIKNYWRTHLRKN 104

Bv_ksge IRKGPWTEQEDLQLVCFV-GLFGD-----RRWDFIAKVS--GLKRTGKSCRLRWVNYLHPGLKRG-KMTPHEEKLVLELHSQWGNRWSRIARRLPGRTDNEIKNYWRTHMRKK 104

Bv_ezhe LKKGPWSAAEDAVLVDYV-KLHGE-----GNWNAVQRNT--GLQRCGKSCRLRWANHLRPNLKKG-AFSSHEERLIIDLHAKLGNKWARISAHLPGRTDNEIKNYWNTRVKRR 104

Bv_ygxg LKKGPWTSAEDAILVEYV-TKHGE-----GNWNAVQKHS--GLSRCGKSCRLRWANHLRPDLKKG-AFTPEEENRIIELHAKMGNKWARMAAELPGRTDNEIKNYWNTRIKRL 104

Bv_zqor IRKGPWKAEEDQVLLNHV-QKYGP-----RDWSSIRSKG--LLQRTGKSCRLRWVNKLRPNLKNGCKFTAEEEKVVIELQAQLGNKWARIATHLPGRTDNDVKNFWSSRQKRL 105

Bv_ohkk KERQRWTTEEDTILGAYV-KQYGP-----REWHLVSQRMNTPLDRDAKSCLERWKNYLKPGIKKG-SLTEEEQRLVIRLQAKHGNKWKKIAAEVPGRTAKRLGKWWEVYKEKQ 106

Bv_zeqy RRIVTWTQEEDDILREQI-SVHGT-----ENWTIIASKFK---DKTTRQCRRRWYTYLNSDFKKG-GWSPEEDMLLCEAQKIFGNRWTEIAKVVSGRTDNAVKNRFSTLCKKK 103

* :* . * : : :.* ** . : . . . * : *.:* :: . ** : : :

**Additional file 1. Multiple alignment of MYB domains of 70 *B. vulgaris* R2R3-MYB proteins.**

ClustalW amino acid sequence alignment. The black arrowheads at the top indicate the conserved, regulary spaced tryptophan (W) or phenylalanine (F) residues. Position numbers at the top correspond to those given in Figure 2.
