# Supplementary material for: Genome-wide identification and characterisation of R2R3-MYB genes in sugar beet (Beta vulgaris)
Source: BMC Plant Biol. 2014 Sep 25;14:249. doi: 10.1186/s12870-014-0249-8 (PMC4180131; doi:10.1186/s12870-014-0249-8)
Supplement: Additional file 2: — Phylogenetic Maximal Parsimony (MP) tree (1000 bootstraps) with MYB proteins from B. vulgaris (Bv), A. thaliana (At) and landmark MYBs from other plants built with MEGA5.2. Bootstrap measures are given at the branches. Clades (and Subgroups) are labelled and indicated by different shades of grey. [file 12870_2014_249_MOESM2_ESM.pdf]

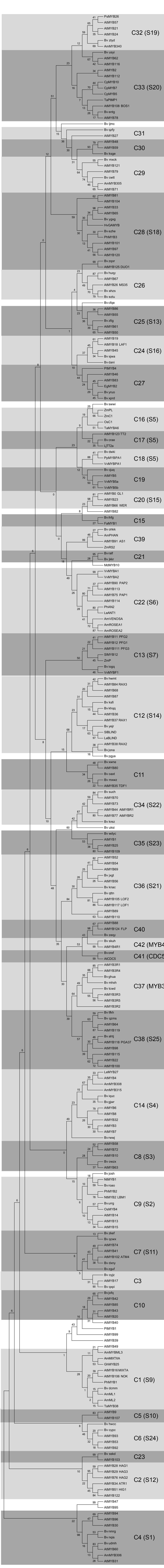

Additional File 2. Phylogenetic Maximal Parsimony (MP) tree (1000 bootstraps) with MYB proteins from *Beta vulgaris (Bv)*, *Arabidopsis thaliana (At)* and other plants.
Bootstrap measures are given at the branches.
