# Supplementary material for: Genome-wide identification and characterisation of R2R3-MYB genes in sugar beet (Beta vulgaris)
Source: BMC Plant Biol. 2014 Sep 25;14:249. doi: 10.1186/s12870-014-0249-8 (PMC4180131; doi:10.1186/s12870-014-0249-8)
Supplement: Additional file 3: — Expression of BvMYB genes in organs of the B. vulgaris reference line KWS2320, deduced from RNA-seq data from the study of Dohm et al. ([ 26 ]). Reads per gene were counted based on BvMYB gene structures. Single count tables for each organ were transformed to normalised data according to the method of Anders and Huber (2010), using correction factors for the different data sets which have been computed as 4.02, 1.25, 3.53, 0.11, 1.47, 0.41 for seedlings, taproot, young leaf, old leaf, inflorescence, seed, respectively. [file 12870_2014_249_MOESM3_ESM.docx]

**Additional file 3. Expression of *BvMYB* genes in organs of the *B. vulgaris* reference line KWS2320, deduced from RNA-seq data from the study of Dohm et al. (2014).**

Reads per gene were counted based on *BvMYB* gene structures. Single count tables for each organ were transformed to normalized data according to the method of Anders and Huber (2010), using correction factors for the different data sets which have been computed as 4.02, 1.25, 3.53, 0.11, 1.47, 0.41 for seedlings, taproot, young leaf, old leaf, inflorescence, seed, respectively.

|  |  |  |  |  | **normalized RNA-seq reads** | | | | | |
| --- | --- | --- | --- | --- | --- | --- | --- | --- | --- | --- |
| **gene ID** | **gene code** | **chr.** | **clade (subgroup)** | **functional assignment** | **seedling** | **taproot** | **young leaf** | **old leaf** | **inflores-cence** | **seed** |
| iquc | Bv1g001230_iquc | 1 | C14 (S4) | metabolism | 349.0 | 97.2 | 285.8 | 940.2 | 44.9 | 316.5 |
| owzx | Bv1g001750_owzx | 1 | C8 (S3) | metabolism | 38.1 | 9.6 | 0.8 | 0 | 62.6 | 108.7 |
| dwki | Bv1g002800_dwki | 1 | C18 (S5) | metabolism | 0 | 0 | 0 | 0 | 44.9 | 0 |
| qxpi | Bv1g006050_qxpi | 1 | C3 | development | 33.6 | 5.6 | 24.9 | 184.3 | 242.9 | 82.2 |
| ksfi | Bv1g014750_ksfi | 1 | C12 (S14) | development | 34.3 | 15.3 | 0 | 0 | 0 | 12.1 |
| zqor | Bv1ug018140_zqor | 1un | C28 (S18) | development | 0 | 0 | 0 | 0 | 12.9 | 4.8 |
| jxgt | Bv1ug021520_jxgt | 1un | C36 (S21) | devel., metab. | 4.7 | 8.8 | 0.8 | 0 | 54.4 | 29 |
| uksi | Bv2g023560_uksi | 2 | C35 (S23) |  | 36.6 | 2.4 | 0.8 | 0 | 25.2 | 19.3 |
| wdyc | Bv2g024650_wdyc | 2 | C35 (S23) |  | 127.1 | 180.7 | 82.6 | 368.7 | 149.7 | 251.3 |
| ihfg | Bv2g027580_ihfg | 2 | C15 | metabolism | 74.6 | 46.6 | 123.1 | 73.7 | 112.3 | 91.8 |
| jkkr | Bv2g027795_jkkr | 2 | C21 | (metabolism) | 0 | 0 | 0 | 0 | 0 | 0 |
| mxck | Bv2g027990_mxck | 2 | C29 |  | 1.2 | 2.4 | 0 | 0 | 0.7 | 0 |
| xprd | Bv2g029260_xprd | 2 | C27 | metabolism | 14.7 | 6.4 | 8.5 | 36.9 | 31.3 | 0 |
| ralf | Bv2g030925_ralf | 2 | C21 | (metabolism) | 0 | 0 | 0 | 0 | 0 | 0 |
| ghua | Bv2g031800_ghua | 2 | C37 (MYB3R) | cell cycle | 446.3 | 799 | 82.1 | 350.3 | 249.7 | 33.8 |
| huqy | Bv2g039110_huqy | 2 | C26 | development | 2.7 | 1.6 | 3.4 | 0 | 46.3 | 4.8 |
| dcmm | Bv2g040720_dcmm | 2 | C1 (S9) | differentiation | 144 | 13.7 | 23.5 | 516.2 | 184.4 | 33.8 |
| mxwz | Bv2g041120_mxwz | 2 | C11 | development | 2.5 | 2.4 | 0 | 0 | 32 | 0 |
| nqis | Bv2ug047120_nqis | 2un | C4 (S1) | defense | 100.5 | 0.8 | 188.2 | 92.2 | 207.5 | 31.4 |
| urrg | Bv3g049510_urrg | 3 | C9 (S2) | defense | 36.1 | 8 | 33.4 | 9.2 | 40.1 | 60.4 |
| hwcc | Bv3g050090_hwcc | 3 | C6 (S24) |  | 13.9 | 53.8 | 0 | 0 | 16.3 | 123.2 |
| cwtt | Bv3ug070140_cwtt | 3un | C29 |  | 8.5 | 45.8 | 0.3 | 0 | 6.1 | 0 |
| cjuq | Bv4g071740_cjuq | 4 | C19 | metabolism | 5 | 7.2 | 11.9 | 18.4 | 52.4 | 9.7 |
| yruo | Bv4g073190_yruo | 4 | C27 | metabolism | 10 | 2.4 | 13 | 9.2 | 121.8 | 4.8 |
| ygxg | Bv4g074860_ygxg | 4 | C28 (S18) | development | 147.5 | 305.9 | 490.5 | 110.6 | 177.6 | 82.2 |
| skuh | Bv4g078900_skuh | 4 | C42 (MYB4R) |  | 506 | 484.2 | 154 | 110.6 | 353.8 | 1128.4 |
| zfig | Bv4g079610_zfig | 4 | C25 (S13) | metabolism | 442.1 | 155.8 | 125.9 | 36.9 | 325.3 | 2.4 |
| oref | Bv4g079670_oref | 4 | C41 (CDC5) | cell cycle | 1586.4 | 2190.5 | 524.1 | 322.6 | 932.2 | 1918.5 |
| josh | Bv4g083815_josh | 4 |  |  | 0 | 0.8 | 0 | 0 | 0 | 0 |
| rwwj | Bv4g084340_rwwj | 4 | C14 (S4) | metabolism | 45.8 | 20.1 | 5.1 | 27.7 | 35.4 | 12.1 |
| xwne | Bv4g091510_xwne | 4 | C11 | development | 141.3 | 123.7 | 141.5 | 101.4 | 82.3 | 41.1 |
| jofq | Bv5g098940_jofq | 5 | C10 |  | 140.3 | 81.1 | 105.3 | 64.5 | 168.7 | 87 |
| sskd | Bv5g100530_sskd | 5 | C23 | development | 0.5 | 1.6 | 0 | 0 | 30.6 | 0 |
| mhxh | Bv5g101320_mhxh | 5 | C37 (MYB3R) | cell cycle | 40.3 | 51.4 | 37.1 | 9.2 | 45.6 | 33.8 |
| zkef | Bv5g107260_zkef | 5 |  |  | 0 | 0 | 0 | 0 | 4.1 | 0 |
| ztyd | Bv5g110930_ztyd | 5 | C32 (S19) | development | 0 | 0 | 0 | 0 | 0 | 0 |
| udmh | Bv5g110960_udmh | 5 | C4 (S1) | defense | 13.2 | 1.6 | 224.4 | 46.1 | 213 | 14.5 |
| tcwd | Bv5g112510_tcwd | 5 | C37 (MYB3R) | cell cycle | 171.4 | 207.2 | 148.6 | 165.9 | 140.2 | 352.8 |
| nmrg | Bv5g115970_nmrg | 5 | C4 (S1) | defense | 237.6 | 16.1 | 115.2 | 811.1 | 216.4 | 210.2 |
| oaxt | Bv5g116880_oaxt | 5 | C11 | development | 0 | 0 | 0 | 0 | 46.3 | 0 |
| tfkh | Bv5g118200_tfkh | 5 | C38 (S25) | development | 3.7 | 0 | 0.3 | 0 | 0.7 | 43.5 |
| ahtj | Bv5g118320_ahtj | 5 | C38 (S25) | development | 0 | 0 | 0 | 0 | 7.5 | 0 |
| cfqe | Bv5g118940_cfqe | 5 |  |  | 23.1 | 52.2 | 15.6 | 0 | 25.9 | 0 |
| roao | Bv5g122000_roao | 5 | C9 (S2) | defense | 243.8 | 122.1 | 19 | 239.6 | 95.3 | 118.4 |
| iogq | Bv5g122370_iogq | 5 | C13 (S7) | metabolism | 104.5 | 2.4 | 26.6 | 0 | 104.1 | 19.3 |
| ijmc | Bv5g123335_ijmc | 5 | C33 (S20) | def., devel. | 0.2 | 0 | 0 | 0 | 0 | 0 |
| ohkk | Bv5ug126300_ohkk | 5un | C39 | development | 1029.7 | 815.8 | 931.4 | 986.3 | 770.9 | 1406.2 |
| knac | Bv5ug126380_knac | 5un | C36 (S21) | devel., metab. | 9.7 | 31.3 | 15 | 36.9 | 44.2 | 9.7 |
| qcwx | Bv5ug126530_qcwx | 5un | C7 (S11) | defense | 5.7 | 12.0 | 0 | 0 | 1.4 | 123.2 |
| such | Bv6g128620_such | 6 | C34 (S22) | def., devel. | 796.1 | 5983.8 | 669 | 10950.1 | 852.6 | 261 |
| hwmt | Bv6g129790_hwmt | 6 | C12 (S14) | development | 102.7 | 199.1 | 4 | 0 | 4.8 | 14.5 |
| oypc | Bv6g136060_oypc | 6 | C6 (S24) |  | 1.5 | 66.6 | 0 | 9.2 | 17 | 4.8 |
| usyi | Bv6g142590_usyi | 6 | C33 (S20) | def., devel. | 45.5 | 33.7 | 79 | 0 | 121.8 | 125.6 |
| qttn | Bv6g154730_qttn | 6 | C36 (S21) | devel., metab. | 8.2 | 0 | 10.2 | 0 | 42.9 | 4.8 |
| zeqy | Bv6g155340_zeqy | 6 | C40 | differentiation | 316.7 | 672.1 | 364.5 | 92.2 | 360.6 | 94.2 |
| yejr | Bv7g162730_yejr | 7 | C12 (S14) | development | 70.9 | 20.1 | 4 | 0 | 4.1 | 106.3 |
| ahzs | Bv7g172570_ahzs | 7 | C26 | development | 0.2 | 1.6 | 0 | 0 | 64.6 | 212.6 |
| eztu | Bv7g172590_eztu | 7 | C26 | development | 0.5 | 0 | 0.3 | 0 | 168.7 | 65.2 |
| qzms | Bv7g174540_qzms | 7 | C38 (S25) | development | 0 | 0 | 0.3 | 0 | 5.4 | 0.0 |
| ksge | Bv7g176420_ksge | 7 | C30 | development | 179.6 | 309.1 | 362.5 | 129.0 | 187.8 | 48.3 |
| qzfy | Bv7ug180860_qzfy | 7un | C31 |  | 10.4 | 1.6 | 1.7 | 0 | 2.0 | 43.5 |
| dxny | Bv8g183050_dxny | 8 | C7 (S11) | defense | 111.2 | 3.2 | 53.8 | 9.2 | 21.1 | 720.0 |
| zguf | Bv8g183060_zguf | 8 | C7 (S11) | defense | 26.6 | 0 | 0 | 0 | 2.0 | 207.8 |
| jona | Bv8g199535_jona | 8 | C12 (S14) | development | 0 | 0 | 0 | 0 | 0 | 0 |
| khqq | Bv8g200250_khqq | 8 | C12 (S14) | development | 77.1 | 16.9 | 0.3 | 0 | 2.0 | 43.5 |
| gjwr | Bv9g216350_gjwr | 9 | C14 (S4) | metabolism | 215.4 | 32.1 | 236.9 | 110.6 | 653.2 | 282.7 |
| krez | Bv9g225930_krez | 9 | C34 (S22) | def., devel. | 212.2 | 2107.8 | 245.7 | 2092.3 | 138.8 | 459.1 |
| ezhe | Bvg229250_ezhe | rnd0020 | C28 (S18) | development | 156.7 | 4.0 | 0.6 | 9.2 | 136.8 | 113.6 |
| crae | Bvg229400_crae | rnd0039 | C17 (S5) | metabolism | 20.2 | 89.9 | 85.5 | 9.2 | 89.8 | 84.6 |
| entg | Bvg229850_entg | rnd0043 | C33 (S20) | def., devel. | 24.1 | 38.5 | 9.6 | 0 | 155.8 | 171.6 |
| swwi | Bvg235150_swwi | rnd0157 |  |  | 0 | 0 | 0 | 0 | 20.4 | 4.8 |
| oyjz | Bvg238960_oyjz | rnd0254 | C3 | development | 0.5 | 0.8 | 0 | 0 | 0 | 0 |
| dani | Bvg239075_dani | rnd0254 | C24 (S16) | development | 0 | 0 | 0 | 0 | 0 | 0 |
| sjwa | Bvg239080_sjwa | rnd0254 | C24 (S16) | development | 0.7 | 2.4 | 0.0 | 0.0 | 0.7 | 0 |
| pgya | Bvg243050_pgya | rnd0446 |  |  | 68.7 | 77.9 | 144.6 | 27.7 | 94.6 | 82.2 |
|  |  |  |  |  |  |  |  |  |  |  |
