# Supplementary material for: Genome-wide identification and characterisation of R2R3-MYB genes in sugar beet (Beta vulgaris)
Source: BMC Plant Biol. 2014 Sep 25;14:249. doi: 10.1186/s12870-014-0249-8 (PMC4180131; doi:10.1186/s12870-014-0249-8)
Supplement: Additional file 7: — Trimming and mapping of RNA-seq reads. (A) Trimming of RNA-seq raw reads using Trimmomatic. SRA: Sequence read archive. (B) Mapping of RNA-seq reads to RefBeet after applying Tophat. [file 12870_2014_249_MOESM7_ESM.doc]

**Additional file 4. Trimming and mapping of RNA-seq reads.**

**A.** Trimming of RNA-seq raw reads using *Trimmomatic*. SRA: Sequence read archive.

| **SRA accession** | **RNA-seq library** | **Sample** | **Read length (bases)** | **Number of input pairs** | **Both surviving** | **Both** | **Only forward** | **Only reverse** | **Dropped** |
| --- | --- | --- | --- | --- | --- | --- | --- | --- | --- |
| SRX287611 | HiF | inflorescence | 100 | 78,981,211 | 64,506,296 | 81.7% | 4.1% | 4.6% | 9.6% |
| SRX287612 | HiF2 | inflorescence | 50 | 89,371,677 | 69,715,500 | 78.0% | 6.5% | 5.6% | 9.9% |
| SRX287609 | 1R_1 | root | 76 | 27,481,944 | 15,749,120 | 57.3% | 9.7% | 12.9% | 20.2% |
| SRX287610 | 1R_2 | root | 36 | 35,989,483 | 26,477,374 | 73.6% | 4.7% | 6.7% | 15.1% |
| SRX287608 | 1B | leaf | 54 | 26,670,031 | 13,654,896 | 51.2% | 12.9% | 11.3% | 24.6% |
| SRX287614 | HiS | seed | 50 | 29,650,038 | 24,040,591 | 81.1% | 5.2% | 5.2% | 8.5% |
| SRX287615 | HiK | seedling | 50 | 76,597,255 | 62,950,703 | 82.2% | 4.4% | 5.4% | 8.0% |
| SRX287613 | HiK2 | seedling | 50 | 86,010,137 | 70,108,661 | 81.5% | 4.7% | 5.2% | 8.6% |

**B.** Mapping of RNA-seq reads to RefBeet after applying *Tophat*.

| **Sample** | **Number of input reads** | **Number of mapped reads** | **Mapped reads %** | **Number of properly paired reads** | **Number of singletons** |
| --- | --- | --- | --- | --- | --- |
| inflorescence | 268,442,341 | 200,094,674 | 74.54 | 147,289,890 | 15,249,324 |
| root | 84,450,452 | 71,817,423 | 85.04 | 57,132,842 | 2,766,363 |
| seed | 48,079,176 | 39,517,367 | 82.19 | 20,623,890 | 885,953 |
| leaf | 27,307,447 | 23,921,502 | 87.60 | 18,066,834 | 1,019,336 |
| seedling | 266,117,964 | 236,727,199 | 88.96 | 163,834,110 | 8,445,981 |
